# Integration of multiple flexible electrodes for real-time detection of barrier formation with spatial resolution in a gut-on-chip system

**DOI:** 10.1038/s41378-023-00640-x

**Published:** 2024-01-24

**Authors:** Mara Lucchetti, Gabriel Werr, Sofia Johansson, Laurent Barbe, Léa Grandmougin, Paul Wilmes, Maria Tenje

**Affiliations:** 1 Luxembourg Centre for Systems Biomedicine (LCSB), University of Luxembourg, Esch-sur-Alzette, L-4362 Luxembourg; 2 Division of Biomedical Engineering, Department of Materials Science and Engineering, Science for Life Laboratory, Uppsala University, 751 21 Uppsala, Sweden; 3 Department of Life Sciences and Medicine, Faculty of Science, Technology and Medicine, University of Luxembourg, Esch-sur-Alzette, L-4362 Luxembourg

**Keywords:** Electrical and electronic engineering, Biosensors, Microfluidics

## Abstract

In healthy individuals, the intestinal epithelium forms a tight barrier to prevent gut bacteria from reaching blood circulation. To study the effect of probiotics, dietary compounds and drugs on gut barrier formation and disruption, human gut epithelial and bacterial cells can be cocultured in an in vitro model called the human microbial crosstalk (HuMiX) gut-on-a-chip system. Here, we present the design, fabrication and integration of thin-film electrodes into the HuMiX platform to measure transepithelial electrical resistance (TEER) as a direct readout on barrier tightness in real-time. As various aspects of the HuMiX platform have already been set in their design, such as multiple compressible layers, uneven surfaces and nontransparent materials, a novel fabrication method was developed whereby thin-film metal electrodes were first deposited on flexible substrates and sequentially integrated with the HuMiX system via a transfer-tape approach. Moreover, to measure localized TEER along the cell culture chamber, we integrated multiple electrodes that were connected to an impedance analyzer via a multiplexer. We further developed a dynamic normalization method because the active measurement area depends on the measured TEER levels. The fabrication process and system setup can be applicable to other barrier-on-chip systems. As a proof-of-concept, we measured the barrier formation of a cancerous Caco-2 cell line in real-time, which was mapped at four spatially separated positions along the HuMiX culture area.

## Introduction

Drug development and basic medical research rely heavily on animal experiments^1^. To optimize the time at which animal experiments are introduced to the development and research pipeline, novel methods are being developed to improve the in vivo resemblance of current in vitro platforms and identify more physiologically relevant methods to study tissues, organs, and their associated diseases. As a results, a new research and development field has emerged that uses microfluidics-based systems for cell cultures, which are referred to as microphysiological systems (MPS) or Organs-on-Chips (OoC)^2–5^. Compared to conventional 2D in vitro cell culture systems and animal models, these systems offer a more advanced and ethical approach and exhibit several key advantages^6^. First, the systems more accurately mimic the physiological aspects of an organ, including cell‒cell interactions and the incorporation of patient-specific cells^7^. Second, specific molecular events can occur and be studied without interference from systemic effects^5^. Finally, the cellular environment can be controlled better with OoCs, as many factors can be regulated, such as medium flow, pH, glucose, and oxygen levels^8,9^.

The intestinal epithelium is composed of specialized cells that are tightly connected to each other to form a permeable barrier^10^. Through the tightness of the intestinal epithelium the passage of harmful substances and microorganisms are blocked while essential nutrients can be absorped^11^. This barrier also plays a crucial role in the prevention of various diseases^12^. Given the importance of the intestinal epithelium in maintaining health, substantial research is being conducted to clarify the effect of external and internal factors on barrier function^13^. The human microbial crosstalk (HuMiX) gut-on-a-chip, which was first described by Shah et al.^14^, is a microfluidic system that can model the intestinal barrier in relation to the intestinal microbiome in a representative manner^15^. Using the HuMiX system, an understanding of the importance of using synbiotics in the therapeutic treatment of colorectal cancer (CRC)^16^ and of the effect of microbiome-derived metabolites in driving CRC development^17^ has been obtained.

When culturing cells over long time periods in a closed organ-on-chip system, such as the HuMiX platform, it is important to continuously monitor cell differentiation in a nonintrusive manner. This ability is an important internal quality control, important for standardization and a direct readout of environmental factors affecting barrier integrity. Transepithelial/endothelial electrical resistance (TEER) readout is used extensively in in vitro models^18^ to assess the permeability of barrier-forming cells, such as intestinal and endothelial cells^9^, as the method is straightforward to integrate and does not interfere with cultured cells during operation. Importantly, TEER provides short response times and good S/N ratios, making it a suitable method for evaluating the effects of drugs, toxins and other substances on the integrity of barriers^19^ and studying the mechanisms underlying barrier dysfunction in various diseases.

Due to the simplicity of integration and operation, TEER measurements have been integrated in OoCs to monitor cell barrier tightness using metal wires^20^ or thin-film microfabricated electrodes^21^. However, the integration of electrodes for TEER in OoCs is challenging due to the large design variability of current OoC systems, which prevents the establishment of standardized electrode layout and fabrication protocols. Moreover, current reports on OoC integrated TEER measurements typically only include data collected from one 4-point measurement across the cell culture area at a single frequency, which is normally an integral measurement representative of the whole biological barrier.

In this work, we address the outstanding challenges of integrating in situ TEER readout in OoCs with more complex designs by patterning thin-film metal electrodes on flexible polyimide tape substrates and carrier foils. More specifically, we address issues encountered as electrodes are positioned in the channel at which the electrode can be freely moved, positioned relative to the channel and fixed in place by the tape (through the tape and carrier foil combination), which occurs before the parts are used in a sterile environment. The tape also helped conform the electrodes to the uneven membrane sections, which can occur in the channel area due to tensions in the membrane, avoiding closed off pockets between the membrane and electrode. Last, the gaskets could conform around the edges of the polyimide tape, forming a tight seal and solving the leakage issue we experienced with wire electrodes or more rigid substrates such as glass cover slips. To collect data from multiple sites across the cell culture area and obtain a more detailed picture of the biological barrier’s function and structure, we integrated 8 electrode pairs to support multiple localized 4-point measurements that are spatially distributed across the cell culture channel. Moreover, to increase information density, we collected data at multiple frequencies using impedance spectroscopy. As a proof-of-concept, we utilized the designed TEER electrodes to measure real-time barrier formation, disruption and recovery of an epithelial cell line in the previously developed HuMiX platform.

## Results and discussion

### Fabrication and integration of multiple individually addressable TEER electrodes into the HuMiX platform

The developed fabrication protocol achieved a process yield of 83%, and the most critical step was related to spinning the resist onto the tape surface where contamination and scratches from the previous process steps may leave fine streaks in the resist layer, causing short circuits between the individual thin-film electrodes. Another challenge with the process was to align the electrodes and the cell culture channels well to obtain the same active area of electrodes in all devices. In our first approach, we placed the empty tape on the membrane and manually aligned a stainless-steel shadow mask directly to the channels for this step; however, we subsequently decided to first shape the electrode on the tape by spin-coating a photoresist to achieve higher quality electrode definition and align the electrodes in a later stage of assembly. Although access to cleanroom facilities is necessary, which is not the case for the shadow-masking approach, this issue was outweighed by the higher throughput achieved using a batch processing method. Another important fabrication step was the addition of an Ar-plasma treatment step before metal sputtering. This step increased the adhesion of electrodes to the carrier foil, allowing it to tolerate the transfer from fluorinated foil to the HuMiX layers. No change in sheet resistance was measured after the electrodes were transferred from the carrier sheet to the HuMiX layers (Fig. S1). This result demonstrates that the bending stresses during transfer did not result in delamination or cracking of the metal layer. Through the transfer foil, the fabrication of the electrodes was separated from HuMiX, greatly increasing the processing options since compatibility with the HuMiX parts was not necessary. Finally, a vital step for realizing these electrodes was to identify relevant material combinations of foils that could withstand the heat and solvent treatments during processing. While electrodes on foil were also an option, the tape was chosen as an essential improvement to the assembly process. Electrodes could be aligned and placed on the HuMiX layers before sterilization, moving that step out of the laminar flow bench. The tape also ensured that no cavities formed between the electrode and the HuMiX layers.

Sequential 4-point measurements on a maximum of eight channels were achieved by interfacing the TEER electrodes with the impedance analyzer through a multiplexer (MUX). The process of acquiring an impedance sweep took less than one minute for the investigated settings, which limits the temporal resolution for TEER monitoring to 8 min when measuring over all eight channels. A time interval of 15 min was chosen for the reported TEER data in this study.

### The integrated electrodes enable localized TEER measurements along the cell culture area

When the electrodes are much smaller than the cell culture membrane, special effort is needed to normalize TEER data, as the TEER value (in Ωcm^2^) will be overestimated and the TEER value will be underestimated when the full cell culture area and electrode area are used, respectively^22^. Geometrical correction has previously been reported for other geometries^23^. The reason for the difference between the apparent measured and true TEER (tTEER) values in this setup is the nonuniform electrical field across the cell layer, which occurs because the electrodes are much smaller than the cell culture area. Moreover, the shape of the electrical field changes as a function of barrier integrity, i.e., with the magnitude of the measured TEER.

**Fig. 1:**
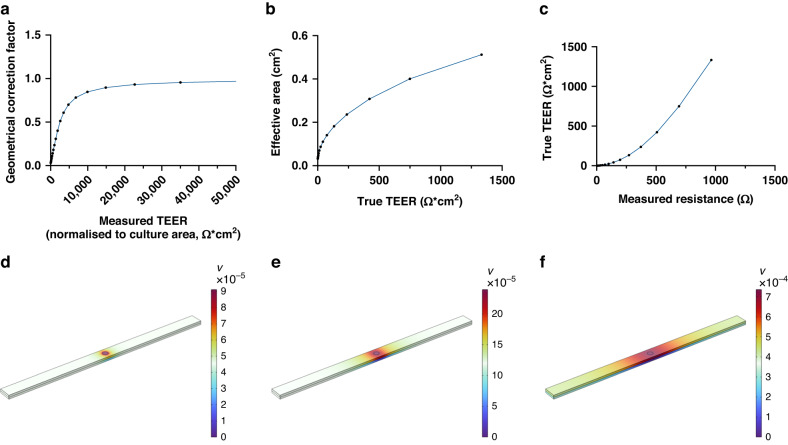
Results from COMSOL modeling to determine the geometrical correction factor for calculating tTEER values for the specific geometry. **a** Geometrical correction factor for different TEER values that has been normalized with the cell culture area. **b** The effective area, i.e., the cell culture area that is being probed for different TEER values by each TEER sensor. The total cell culture area is 2.7 cm^2^. **c** True TEER value vs. measured resistance. **d**–**f** The potential field in the microfluidic channel, which is a good indication of the shape and size of the probed area for **d** TEER = 10 Ωcm^2^, **e** TEER = 100 Ωcm^2^, **f** TEER = 1000 Ωcm^2^

For our specific geometry and using the cell culture area as the reference area, the simulations generated a geometrical correction factor that is small for low barrier integrity and rapidly increases as the TEER (barrier integrity) increases. The correction factor asymptotically increases toward 1, indicating that the electrical field is uniformly distributed for very high TEER values (Fig. 1a). In this HuMiX geometry and for TEER values ranging from 100 to 1000 Ωcm^2^, the geometrical correction factors are 0.16 and 0.45, respectively. Based on these simulations, the effective area is calculated as the area, which gives the true TEER when multiplied by the measured resistance after the background is subtracted (tTEER = effective area*(measured resistance − background resistance)). This value indicates the size of the area being probed in the cell layer (Fig. 1b). Figure 1c shows the nonlinear relationship between the measured resistance and tTEER. The probed area for different TEER values is displayed in Fig. 1d–f and shows that the measurements are more localized for smaller TEER values. Importantly, the simulation shows that we can measure TEER locally in this geometry for mid-range TEER values up to 100 Ωcm^2^.

### Barrier formation, disruption and recovery can be continuously monitored with integrated TEER sensors

**Fig. 2:**
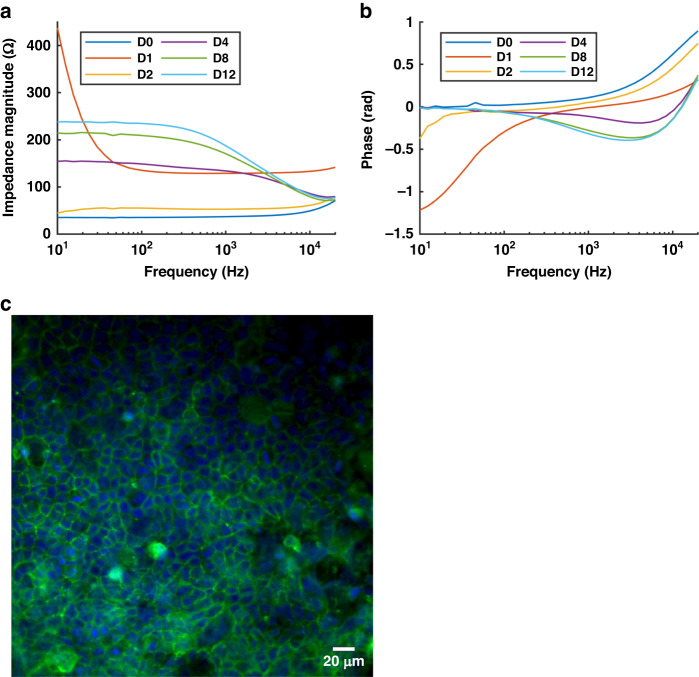
Characterization of the TEER measurements in the HuMiX. **a** Impedance magnitude and **b** phase recorded for 12 days of Caco-2 cell culture inside HuMiX. **c** Immunofluorescence staining of the epithelial Caco-2 barrier grown in the HuMiX for 12 days. Cell nuclei were stained with DAPI (blue) and the tight junction protein occludin was stained with Alexa Fluor 488 (green). The measurements were taken at electrode position 2 (~29 mm from the inlet of the device)

To monitor cell barrier formation, Caco-2 cells were seeded in the epithelial HuMiX chamber, and 4-point impedance readouts were obtained from the integrated Pt electrodes between Day 1 and Day 12 post cell seeding. Example impedance data (impedance magnitude and phase) are displayed in Fig. 2a, b. Immediately after seeding, the cells exhibit a different morphology (more rounded) (Fig. S2a) compared to later time points, as they start attaching to the membrane and form tight junctions between each other (Fig. S2b). This explains why the TEER values at this time point (D0) are very similar to those of naked membranes. The barrier is established during the first few days, and during this period, the impedance does not display the typical curve shape (D1). This is probably because the cell layer reaches a mature state after a few days, as reported by Marrero et al.^24^. From Day 2, the barrier is established, and the characteristic impedance data are observed with a plateau for low frequencies and a dip in the phase (D2–D4), in which the magnitude continues to increase as a tighter barrier is formed (D8–D12). TEER measurements in Transwell cultures showed a similar timeline for barrier formation (Fig. S4a).

On Day 12, the HuMiX was opened to analyze Caco-2 confluency via immunohistochemical staining and microscopy. In accordance with the impedance data from Fig. 2a, b, staining of the tight junction protein occludin after 12 days of culture in the HuMiX system demonstrated a confluent barrier (Fig. 2c). Importantly, Fig. 2c shows a confluent epithelial layer at the same position as the corresponding electrode used to measure impedance.

The formation of a tight cellular barrier is characterized by an increase in impedance in the low-frequency range. Quantitative TEER values can be deduced as an increase in the lower frequency range compared to background measurements without cells (see Eq. 1). With this method, the normalized TEER value for the data in Fig. 2 stabilized at approximately 75 Ωcm^2^. A plot showing the TEER values of this experiment at all time points is provided in the SI, Fig. S3.

**Fig. 3:**
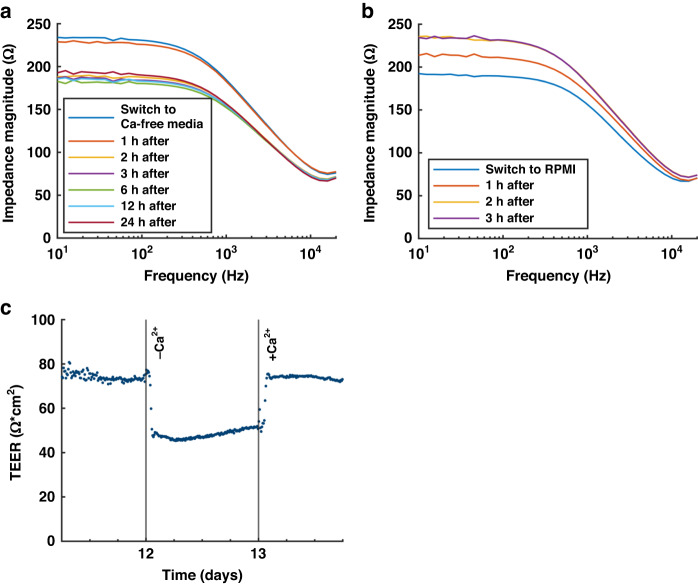
Barrier disruption experiments. **a** Impedance recorded before and up to 24 h after calcium was removed from the cell media. **b** Impedance recorded up to 3 h after replenishing the cell medium with calcium. **c** Normalized TEER as a function of time after seeding, with measurements taken every 15 min. Calcium-free cell media was introduced after 12 days with ordinary cell culture media (RPMI) being reintroduced after 1 day. The measurements were taken at electrode position 2 (~29 mm from the inlet of the device)

Calcium is critical for maintaining cellular tight junctions and ultimately a tight barrier^25,26^. To evaluate whether the presented system can measure Caco-2 barrier disruption, we depleted the cell media of the HuMiX chip of calcium. Removing calcium from the cell media caused a rapid drop in impedance within one hour (Fig. 3a), which is similar to previous reports^27^. When calcium was added to the cell media again, the impedance returned to the initial values within two hours (Fig. 3b). The increase in impedance when calcium was replenished can be explained by a recovery in barrier tightness. Using Eq. 1, the TEER values during the experiment were calculated, as shown in Fig. 3c. Barrier recovery was also observed for the Transwell cultures (Fig. S3b).

### Spatially resolved TEER measurements show barrier nonuniformity along the cell culture area

Due to the integration of multiple TEER sensors, which are individually connected to the impedance analyzer via the MUX, the presented system can measure local TEER at four different positions along the cell culture membrane, which has not previously been reported for other OoCs. To investigate this further, TEER data were extracted for a total of 12,500 impedance sweeps from three different experiments (data not shown). Although the same procedure was followed for all three experiments, there were substantial differences between the TEER data collected, which reflects the varied nature of cell growth in microfluidic systems. For example, despite the addition of bubble traps, there is always a risk of bubbles in microfluidic systems^28^. When a bubble partly or fully blocks an electrode, the impedance increases. In general, bubbles generate a much higher shift in the impedance magnitude than the cell barrier. Moreover, the readout changes more rapidly and exhibits more noise. As opposed to the cell barrier formation, bubbles cause a significant shift in the impedance magnitude also at high frequencies. For the following analysis, a filter was applied where a change in the high-frequency impedance magnitude of more than 200 Ω was used to identify bubbles. These data were subsequently omitted.

**Fig. 4:**
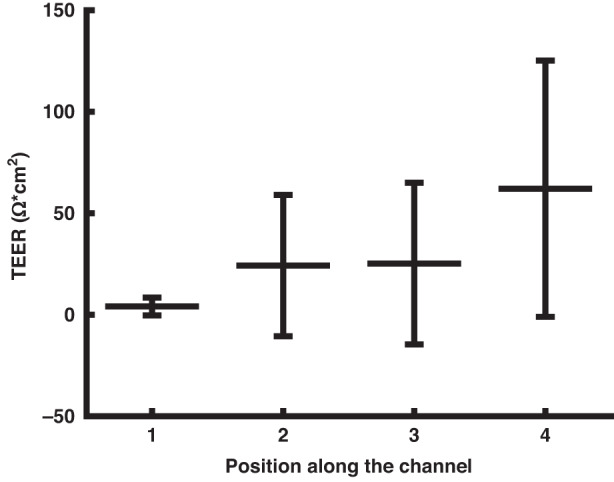
Average TEER values over 10 days for a Caco-2 barrier at four different positions along the cell culture channel. The total length of the channel is 68.4 mm and the respective electrodes are located 22.9, 29.1, 39.3, and 45.5 mm away from the inlet, respectively. The data is normalized by subtracting the background. Mean ± SD of three independent replicates is shown. Position 2 and 3 show no significant difference (confidence interval 66%), all other positions are significantly different to each other (confidence interval 99.99%)

The TEER data from the three experiments were plotted according to their respective positions along the channel in Fig. 4. The results show that the TEER values tended to be higher at areas closer to the outlet of the chip. In particular, the TEER values are lower at position 1, which was closest to the inlet of the chip. The spatially resolved TEER measurements indicate that the barrier integrity is generally poor near the inlet compared to the outlet. Although we might think of the cell culture area as uniform, there are several conditions that can change along the channel, such as chemical gradients, flow lines, temperature and pressure. Given that the cell seeding strategy used in this study, and many other OoCs, consists of manually pushing the cell suspension through the epithelial chamber, the initial cell distribution may be uneven, which leads to the formation of an uneven barrier along the channel length. Additionally, bubbles introduced in the system pose a risk of disrupting the barrier locally, highlighting that the barrier integrity may be nonuniform in other microfluidic systems as well; in addition, single-point TEER measurements in OoCs risk provide inaccurate results on barrier integrity. Furthermore, the information supports the future development of OoCs with multiple integrated electrodes for localized TEER measurements; these OoCs could help researchers more accurately study the response of biological barriers exposed to external stimuli in the form of mechanical forces or chemical compounds.

## Conclusion

In this work, we designed thin-film electrodes and demonstrated their ability to perform real-time impedance measurements along the culture area of HuMiX. In addition, through the multiplexer system, data can be collected from multiple positions within the HuMiX device. Simulations of the electrical field and current distributions in the HuMiX geometry show that local TEER data can be collected in this device for the relevant ranges and that geometrical correction is necessary when normalizing the TEER values. We monitored barrier formation of the Caco-2 cell line over 12 days along the HuMiX membrane area. The recorded impedance data indicated that the barrier integrity was not uniform along the cell culture area. Through the presented fabrication method, the electrode design can be specifically configured and integrated in various OoCs, even with more complex system designs and multiple layers. Thus, this study paves the way for standardized impedance measurements over large and uneven surface areas, providing spatially resolved critical information on barrier integrity.

## Materials and methods

### System design

**Fig. 5:**
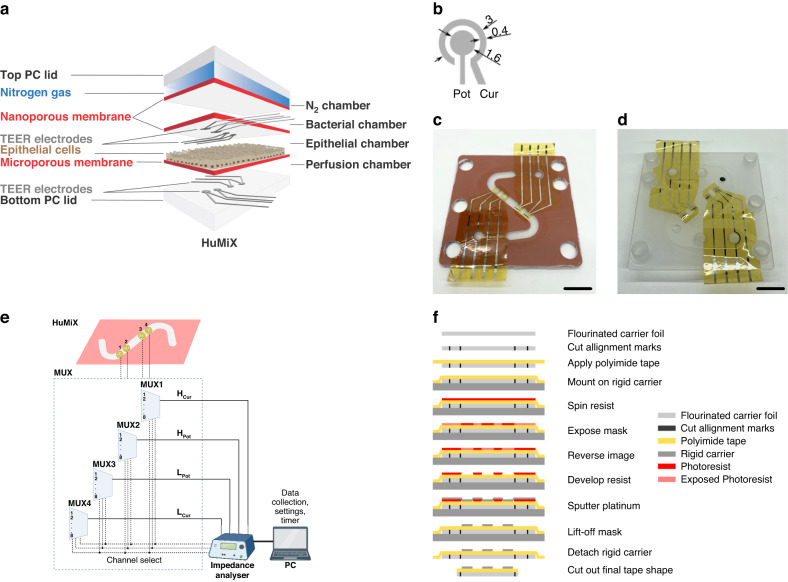
**a** Schematic diagram of HuMiX with the integrated electrodes. **b** Design of the electrode pair used for TEER measurements, units in mm. For each HuMiX device, four electrode pairs were positioned on the bottom side of the HuMiX gasket (**c**) and four electrode pairs were placed on the PC lid (**d**). Scale bar = 1 cm. **e** Schematic of multiplexer connection. MUX 1–4 each switch one of the four measurement connections between up to 8 channels. Their channel selection is connected in parallel to the auxiliary outlets of the impedance analyzer so all four MUX switches are at the same channel. Image created with BioRender. **f** Fabrication process of the TEER electrodes

The HuMiX 3.0 setup was used (Fig. 5a). In comparison to the previously described HuMiX 2.0 and 2.1 versions^16,17^, the HuMiX 3.0 gaskets and polycarbonate (PC) lids are enclosed by metal clamps (stainless steel) to facilitate reproducible clamping alongside fast and easy assembly and disassembly (Fig. S5). The HuMiX 3.0 top chamber was perfused with nitrogen (N_2_) gas (0.1 L/min flow rate), establishing an anoxic environment (~0.4–0.6% O_2_) for the cultivation of anaerobic bacteria in the microbial chamber. However, for the experiments described in this paper, the microbial chamber did not contain bacteria. The top and bottom PC lids enclose silicone rubber gaskets (0.79-mm-thick, custom-made design, Auer Precision Co., Mesa, Arizona, USA), which are attached to semipermeable PC membranes. The separation between the top N_2_ and the microbial chambers as well as the separation between the microbial and epithelial chambers are created using a nanoporous membrane (50 nm, #WHA111703, Merck Milipore, Hoeilaart, Belgium) to allow gas diffusion and media component diffusion, respectively. The third chamber, which harbors epithelial cells, is separated from the fourth/bottom chamber (also called the perfusion chamber) by a microporous membrane (1 µm, #WHAT10418718, VWR, Leuven, Belgium). The epithelial membrane was coated with collagen (rat tail collagen I coating solution, #122-20, Merck Millipore, Hoeilaart, Belgium) to facilitate the attachment of the seeded Caco-2 cell line. The perfusion chamber contained oxic growth medium to supply oxygen and nutrients to the human cells. The previously described spiral-shaped membrane was replaced by a Z-shaped membrane (custom-made design, NTConcept, Palaiseau, France) for the HuMiX used in this study. The purpose of designing a Z-shaped membrane was to (i) reduce the cell surface area and thus the cell seeding number, (ii) create a straight channel and thus reduce bubble formation, and (iii) facilitate electrode insertion.

The Z-shaped microchannels have a length of 68.4 mm, width of 4 mm and height of 0.79 mm and create a chamber volume of 213.2 μl for the top and bottom channels and 261.7 μl for the middle channel. To assemble the system, the gaskets were precisely aligned and sandwiched between the top and bottom PC lid enclosures, and the metal clamp was closed. Figure S4 shows all parts of HuMiX. Autoclavable bubble traps were used (95 μl, #LVF-KBT-M-A, Darwin Microfluidics, Paris, France) to reduce bubble formation in the HuMiX during operation.

### Electrode fabrication

Each TEER sensor comprises two electrode pairs designed in a concentric layout (Fig. 5b), where the outer ring (Cur) provides the excitation current and the inner disk (Pot) measures the voltage signal together with a corresponding electrode pair on the opposite side of the cell layer. TEER values are read out in a 4-point measurement configuration. The size of the electrodes was chosen to maximize fabrication yield, compensate for tolerances during assembly and ensure that the full electrode area is inside the microfluidic channel. A total of 8 electrode pairs were prepared, with four electrode pairs positioned below the epithelial cell layer (Fig. 5c) and another four positioned above it (Fig. 5d); these electrodes were distributed along the HuMiX channels, thereby allowing for read-out along almost the entire cell culture area (Fig. 5e) at spatially separated points. To integrate TEER electrodes in the existing HuMiX system, two additional 55 µm-thick polyimide tape layers were included in the gasket stack above and beneath the epithelial layer (Fig. S4). Figure 5f shows a schematic of the electrode fabrication processes. To handle and align the electrodes, tape (SSA-KAPT0100, 3DJAKE, Paldau, Austria) was applied to a fluorinated foil carrier (SILFLU S 50M 1R88001 CLEAR, Siliconature, Godega di Sant’Urbano, Italy) that included alignment marks, peeling cuts, and a 4-in. wafer shape cut into it with a desktop cutter (CAMM-1 GS-24, Roland DG Corporation, Hamamatsu, Japan). The fluorinated carrier foil was attached to a rigid 700 µm-thick glass wafer to keep the tape flat during processing. To pattern the electrodes on the fluorinated carrier foil, a standard lift-off process was used where a 1.7 µm thin layer of photoresist (MICROPOSIT S1813, micro resist technology GmbH, Berlin, Germany) was first applied to the tape. After the photoresist was soft baked at 100 °C for 2 min, the photoresist was exposed to UV light to pattern the electrodes and an image reversal step was performed (YES-58TA-E, Yield Engineering Systems, Fremont, CA, USA), the photoresist was developed (Microposit™ Developer 351, Micro Resist Technology GmbH, Berlin, Germany). The metal layers, 3 nm Ta and 150 nm Pt, were deposited using a table-top sputter coater (Q300TD, Quorum, Laughton, UK) after a cleaning process that involved a combined Ar/O_2_ plasma treatment (PE-100, Plasma Etch, Inc., Carson City, NV, USA). Finally, the sacrificial photoresist mask was removed with a 5 min acetone ultrasound bath followed by a 1 min isopropanol bath. For successful electrode fabrication, no short-circuiting was detected between any of the electrodes and the connector to measurement area was continuous, which was tested with a multimeter (Fluke 113, Fluke Corporation, Everett, WA, USA). In the final step, the tape was cut to shape on a cutter plotter using the alignment marks of the carrier foil before being sterilized and mounted into the HuMiX.

To achieve a glue and solder-free electrode connection, custom-printed circuit boards (PCBs) were fabricated with pogo-pins to connect to the thin-film Pt electrodes (Fig. S4). The pogo-pins were clamped onto the electrodes with two M2 screws, in which the additional layer facilitated alignment and provided a strain release for the connection cables. Easy connection/disconnection of the HuMiX chips was enabled by USB-connectors between the small PCB and the measurement set up.

### Cell culture

The human epithelial colorectal cell line Caco-2 (passage 10–14) (ACC 169, DSMZ, Braunschweig, Germany) was maintained at 37 °C in a 5% CO_2_ incubator in RPMI medium (RPMI 1640, GlutaMAX Supplement, HEPES, #72400047, Thermo Fisher Scientific, Merelbeke, Belgium) supplemented with 10% fetal bovine serum (FBS HI, #10500-064, Thermo Fisher Scientific) and 1% penicillin–streptomycin (#15140122, Thermo Fisher Scientific) until use and passaged at 80–100% confluency. TrypLE (TrypLE Express Enzyme (1X), phenol red, #12605010, Thermo Fisher Scientific) was used for Caco-2 detachment. The cells were verified monthly for mycoplasma contamination using the MycoAlert® Mycoplasma detection kit (#LT07–318, Lonza, Basel, Switzerland).

### Experimental design and impedance spectroscopy measurements

In this study, three independent experiments were implemented. Control measurements were performed on either an empty channel or a channel filled only with culture media at the start of each experimental series. Approximately 300,000 Caco-2 cells were seeded on the collagen-coated epithelial membrane, which was previously primed with media. Three hours postseeding, a continuous flow in the second (bacterial) and fourth (perfusion) channels was started at a flow rate of 16 μL/min (0.5 rpm, Peristaltic pump, #205 S and #205CA16, Watson-Marlow Fluid Technology Solutions, Zwijnaarde, Belgium) to supply the Caco-2 cells with media via diffusion across the membrane. Media perfusion was maintained throughout the experiment. Bubble traps were connected to the bacterial and perfusion channels of HuMiX to reduce bubble formation. The cells were cultured in HuMiX for a duration of 12 days. For the barrier disruption experiments, normal cell media was replaced with calcium-free media (DMEM, high glucose, no glutamine, no calcium, #21068028, Thermo Fisher Scientific) for 24 h. To support cell barrier recovery, calcium-free media was exchanged with RPMI media again. All measurements were taken with the HuMiX system inside an incubator at 37 °C, and the standard culture protocol used to measure the Caco-2 barrier tightness was not interrupted.

An impedance analyzer (MFIA, Zürich Instruments, Zürich, Switzerland) was used to measure the impedance spectrum of the Caco-2 cell layer over time. The impedance was recorded at 51 logarithmically spaced frequencies between 10 Hz and 500 kHz every 15 min using Pt thin-film electrodes integrated in the HuMiX system above and below the cell layer. To automate the measurements and connect multiple electrodes to the impedance analyzer, four MUXs (MUX36S08EVM-PDK, Texas Instruments, Dallas, TX, USA) were used, allowing up to eight TEER sensors to be connected. The MUX was powered and controlled in parallel by the auxiliary channels of the impedance analyzer (Fig. 5e). In this setup, four TEER sensors per HuMiX were connected, allowing for sequential readout of four localized positions in two parallel HuMiX systems. The settings for the impedance analyzer, the measurement interval, the electrode selection and data collection were controlled using a MATLAB user interface (version MATLAB R2020a, The MathWorks, Inc., Natick, MA, USA).

### Normalization of TEER values

A finite element method was used to calculate the effective area and the geometrical correction factor (GCF) for normalizing the TEER value with the cell culture area (COMSOL Multiphysics, AC/DC module). The parameters and the equations used for the simulation and postprocessing are summarized in Table S1, and the geometry is displayed in Fig. S6. Details on the simulation are found in the supplemented COMSOL report. The geometry was a simplified version of HuMiX using a straight channel with a total cell culture area of 2.7 cm^2^ and TEER sensors with an area of 2 mm^2^ each. The cell layer was modeled for different cell layer conductivities, swept from 7.5 × 10^−5^ S/m to 7.5 S/m, corresponding to TEER values between 0 and 1300 Ωcm^2^.

### Analysis of impedance data

Quantitative TEER was deduced as the difference between the low- and high-frequency impedance magnitude (100 Hz and 50 kHz), and the background measurement with cell media obtained 6 h before seeding was subtracted from that value (mean of 10 measurements at each specific TEER sensor); see Eq. 1. With *Z* being the impedance and in subscript the corresponding frequency and *bg* standing for background measurements, and *GCF*, the geometrical correction factor is obtained from the simulations to normalize the TEER value.

1 $${\rm{TEER}}=\left(\left|{Z}_{100\,{\rm{Hz}}}\right|-\left|{Z}_{50\,{\rm{kHz}}}\right|-\left(\left|{Z}_{100\,{\rm{{Hz}}},{bg}}\right|-{\rm{|}}{Z}_{50\,{\rm{{kHz}}},{bg}}{\rm{|}}\right)\right)\cdot {\rm{GCF}}\cdot {\rm{cell}}\,{\rm{culture}}\,{\rm{area}}$$

The validity of this approach was evaluated by fitting a circuit model to the impedance data (see Fig. S7).

### Transwell experiments

Corning Transwell polycarbonate membrane cell culture inserts (#CLS3401, Merck Millipore, Hoeilaart, Belgium) were used to measure TEER in a 12-well plate. First, Caco-2 cells were seeded (1.2 × 10^5^ cells/insert), and the medium from the apical and basolateral compartments was exchanged every second day. An ERS-2 volt-ohm meter (MERS00002, Merck Millipore, Hoeilaart, Belgium) and STX01 chopstick electrodes (MERSSTX01, Merck Millipore, Hoeilaart, Belgium) were introduced into the well to measure the TEER of the Caco-2 layer. TEER was measured for a total duration of 21 days every second or third day starting from Day 10. Barrier disruption was performed when the Caco-2 cells were fully confluent by exchanging the RPMI media with DMEM. TEER values were calculated by subtracting the TEER value from a cell-free insert and correcting to the cell surface area (1.13 cm^2^).

### Microscopy

Immunofluorescence staining of the tight junction protein occludin was performed using an occludin monoclonal antibody conjugated to Alexa Fluor 488 (#331588, Thermo Fisher Scientific) to confirm barrier formation. At the end of each experiment, the HuMiX was opened, and the epithelial membrane was washed with PBS. The cells were then fixed with 4% paraformaldehyde for 15 min and blocked with 5% BSA for 1 h at room temperature. The cells were labeled with occludin antibody diluted in 4% BSA for 3 h at room temperature, and nuclei were stained overnight with Fluoroshield^TM^ with DAPI (F6057, Merck Milipore, Hoeilaart, Belgium). Both occludin and cell nuclei were visualized using an inverted fluorescence microscope (IX83, Olympus, Antwerpen, Belgium) and CellSens Dimension software for image analysis to identify cell distribution and barrier confluency along the HuMiX channel.

### Supplementary information

COMSOL report

Supplementary Information
