# Supplementary material for: Integration of multiple flexible electrodes for real-time detection of barrier formation with spatial resolution in a gut-on-chip system
Source: Microsyst Nanoeng. 2024 Jan 24;10:18. doi: 10.1038/s41378-023-00640-x (PMC10805851; doi:10.1038/s41378-023-00640-x)
Supplement: Supplementary file 1 — COMSOL report [file 41378_2023_640_MOESM1_ESM.docx]

[
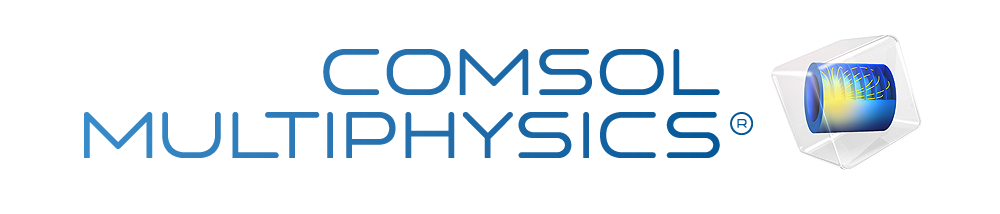
](https://www.comsol.com/)

Humix gcf 3d

| Report date | Jun 16, 2023, 10:47:27 AM |
| --- | --- |

Contents

[1. Global Definitions](#cs2307927)

[1.1. Parameters](#cs8395294)

[1.2. Shared Properties](#cs2912297)

[2. Component 1](#cs1250726)

[2.1. Definitions](#cs8049070)

[2.2. Geometry 1](#cs3899956)

[2.3. Materials](#cs6182877)

[2.4. Electric Currents](#cs9870392)

[2.5. Mesh 1](#cs7898782)

[3. Study 1](#cs2545333)

[3.1. Parametric Sweep](#cs5685825)

[3.2. Stationary](#cs7679031)

[3.3. Solver Configurations](#cs7838953)

[4. Results](#cs7872289)

[4.1. Datasets](#cs8627474)

[4.2. Derived Values](#cs2336072)

[4.3. Plot Groups](#cs2432197)

1. Global Definitions

| Date | May 26, 2023, 11:40:01 AM |
| --- | --- |

Global settings

| Name | Humix gcf 3d.mph |
| --- | --- |
| Path |  |
| Version | COMSOL Multiphysics 6.1 (Build: 252) |
| Unit system | SI |

Used products

| CAD Import Module |
| --- |
| AC/DC Module |
| COMSOL Multiphysics |

Computer information

| CPU | Intel64 Family 6 Model 158 Stepping 9, 4 cores, 15.85 GB RAM |
| --- | --- |
| Operating system | Windows 10 |

- 1. Parameters

Parameters 1

| **Name** | **Expression** | **Value** | **Description** |
| --- | --- | --- | --- |
| channel_w | 6.75 [cm] | 0.0675 m |  |
| channel_d | 4 [mm] | 0.004 m |  |
| channel_h | 1222 [um] | 0.001222 m |  |
| electrode_r | 800 [um] | 8E−4 m |  |
| barrier_h | 10 [um] | 1E−5 m |  |
| barrier_g | 0.15 [S/m] | 0.15 S/m |  |

- 1. Shared Properties
     1. Default Model Inputs

| Tag | cminpt |
| --- | --- |

1. Component 1

| Date | May 26, 2023, 11:07:47 AM |
| --- | --- |

Settings

| **Description** | **Value** |
| --- | --- |
| Unit system | Same as global system (SI) |
| Geometry shape function | Automatic |

Spatial frame coordinates

| **First** | **Second** | **Third** |
| --- | --- | --- |
| x | y | z |

Material frame coordinates

| **First** | **Second** | **Third** |
| --- | --- | --- |
| X | Y | Z |

Geometry frame coordinates

| **First** | **Second** | **Third** |
| --- | --- | --- |
| Xg | Yg | Zg |

Mesh frame coordinates

| **First** | **Second** | **Third** |
| --- | --- | --- |
| Xm | Ym | Zm |

- 1. Definitions
     1. Coordinate Systems

#### Boundary System 1

| Coordinate system type | Boundary system |
| --- | --- |
| Tag | sys1 |

Coordinate names

| **First** | **Second** | **Third** |
| --- | --- | --- |
| t1 | t2 | n |

- 1. Geometry 1


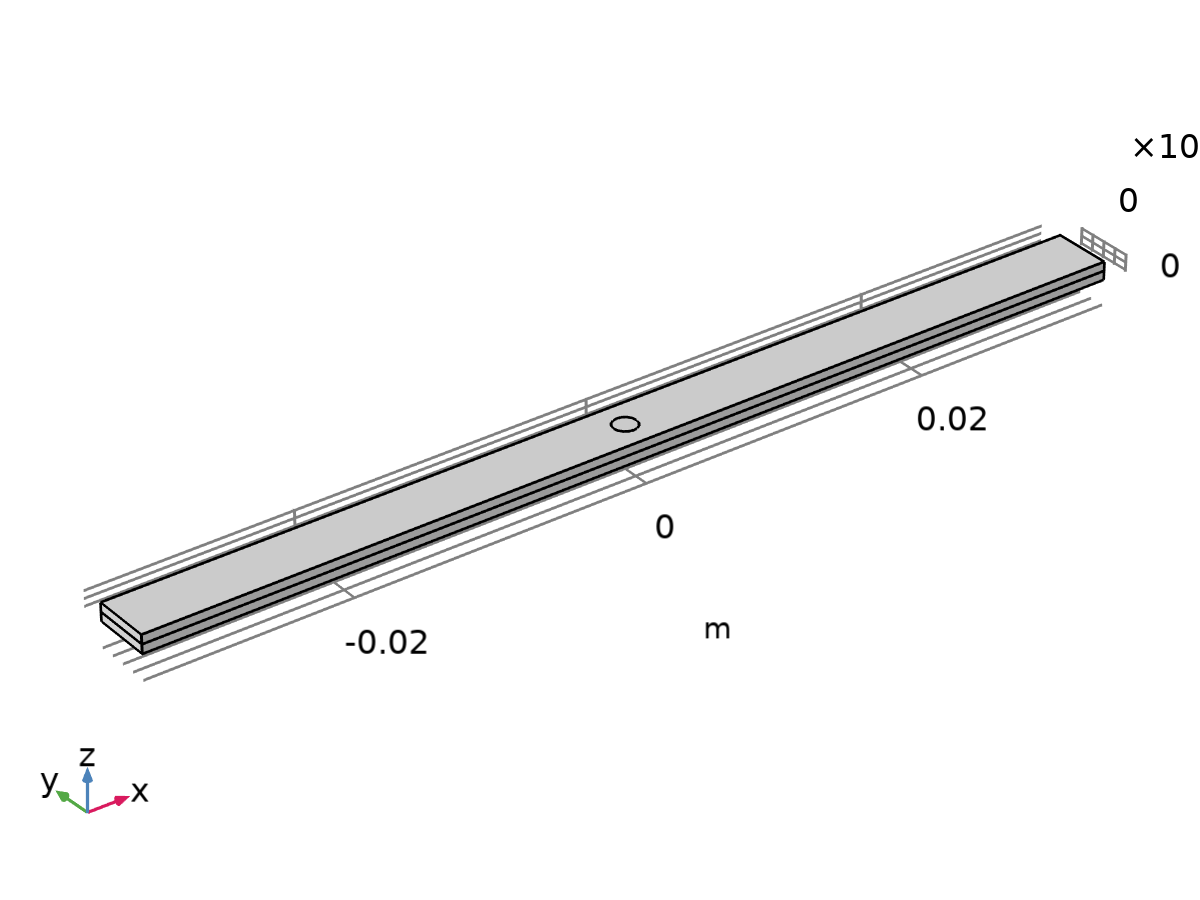


Geometry 1

Units

| Length unit | m |
| --- | --- |
| Angular unit | deg |

Geometry statistics

| **Description** | **Value** |
| --- | --- |
| Space dimension | 3 |
| Number of domains | 3 |
| Number of boundaries | 18 |
| Number of edges | 36 |
| Number of vertices | 24 |

- - 1. Block 1 (blk1)

Position

| **Description** | **Value** |
| --- | --- |
| Position | {0, 0, 0} |
| Base | Center |

Axis

| **Description** | **Value** |
| --- | --- |
| Axis type | z - axis |

Size and shape

| **Description** | **Value** |
| --- | --- |
| Width | channel_w |
| Depth | channel_d |
| Height | channel_h |

- - 1. Cylinder 1 (cyl1)

Position

| **Description** | **Value** |
| --- | --- |
| Position | {0, 0, -channel_h/2} |

Axis

| **Description** | **Value** |
| --- | --- |
| Axis type | z - axis |

Size and shape

| **Description** | **Value** |
| --- | --- |
| Radius | electrode_r |
| Height | channel_h |

- - 1. Union 1 (uni1)

Compose

| **Description** | **Value** |
| --- | --- |
| Keep interior boundaries | Off |

- - 1. Block 2 (blk2)

Position

| **Description** | **Value** |
| --- | --- |
| Position | {0, 0, 0} |
| Base | Center |

Axis

| **Description** | **Value** |
| --- | --- |
| Axis type | z - axis |

Size and shape

| **Description** | **Value** |
| --- | --- |
| Width | channel_w |
| Depth | channel_d |
| Height | barrier_h |

- 1. Materials
     1. Cell Media


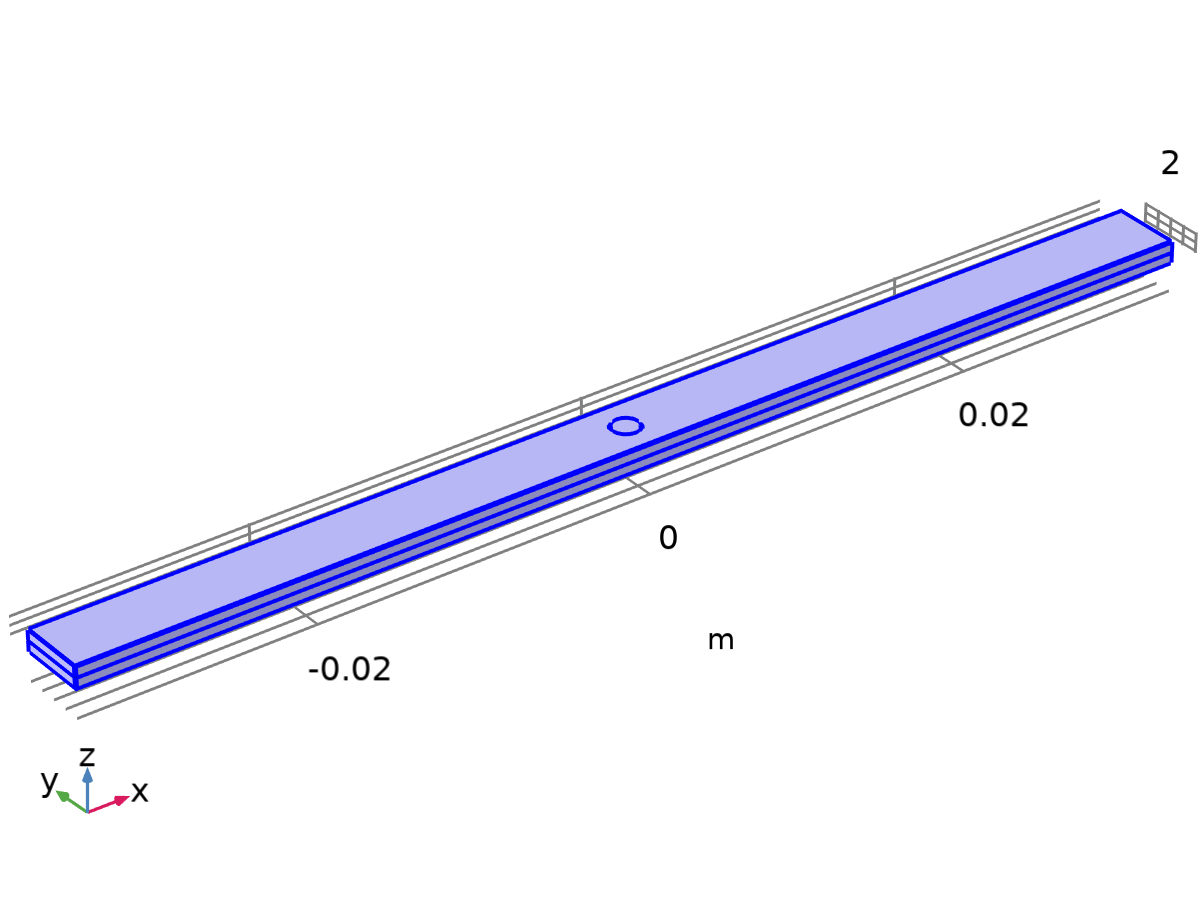


Cell Media

Selection

| Geometric entity level | Domain |
| --- | --- |
| Selection | Geometry geom1: Dimension 3: All domains |

Material parameters

| **Name** | **Value** | **Unit** | **Property group** |
| --- | --- | --- | --- |
| Electrical conductivity | 7.5 | S/m | Basic |
| Relative permittivity | 78 | 1 | Basic |

Basic

| **Description** | **Value** | **Unit** |
| --- | --- | --- |
| Electrical conductivity | 7.5 | S/m |
| Relative permittivity | 78 | 1 |

- - 1. Cell Layer


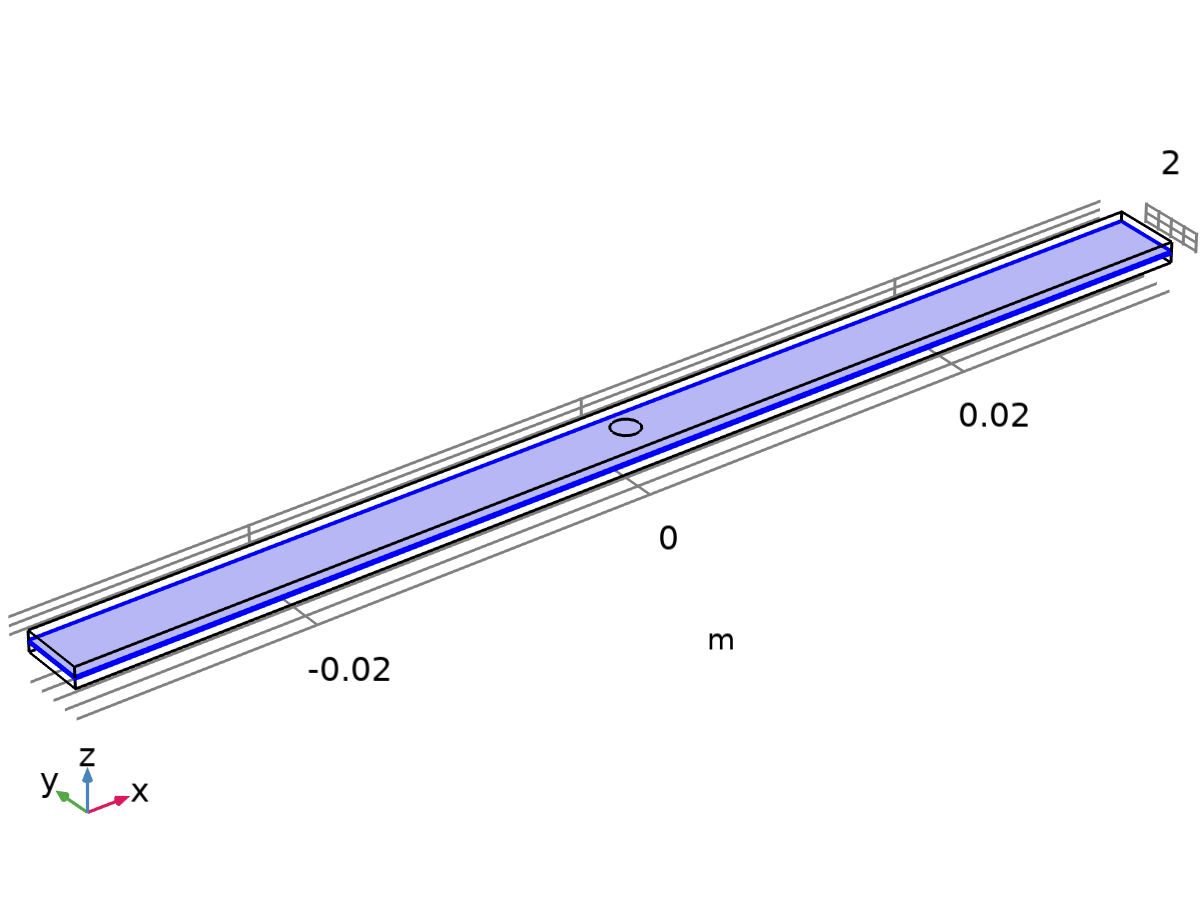


Cell Layer

Selection

| Geometric entity level | Domain |
| --- | --- |
| Selection | Geometry geom1: Dimension 3: Domain 2 |

Material parameters

| **Name** | **Value** | **Unit** | **Property group** |
| --- | --- | --- | --- |
| Electrical conductivity | barrier_g | S/m | Basic |
| Relative permittivity | 78 | 1 | Basic |

Basic

| **Description** | **Value** | **Unit** |
| --- | --- | --- |
| Electrical conductivity | barrier_g | S/m |
| Relative permittivity | 78 | 1 |

- 1. Electric Currents

Used products

| AC/DC Module |
| --- |
| COMSOL Multiphysics |


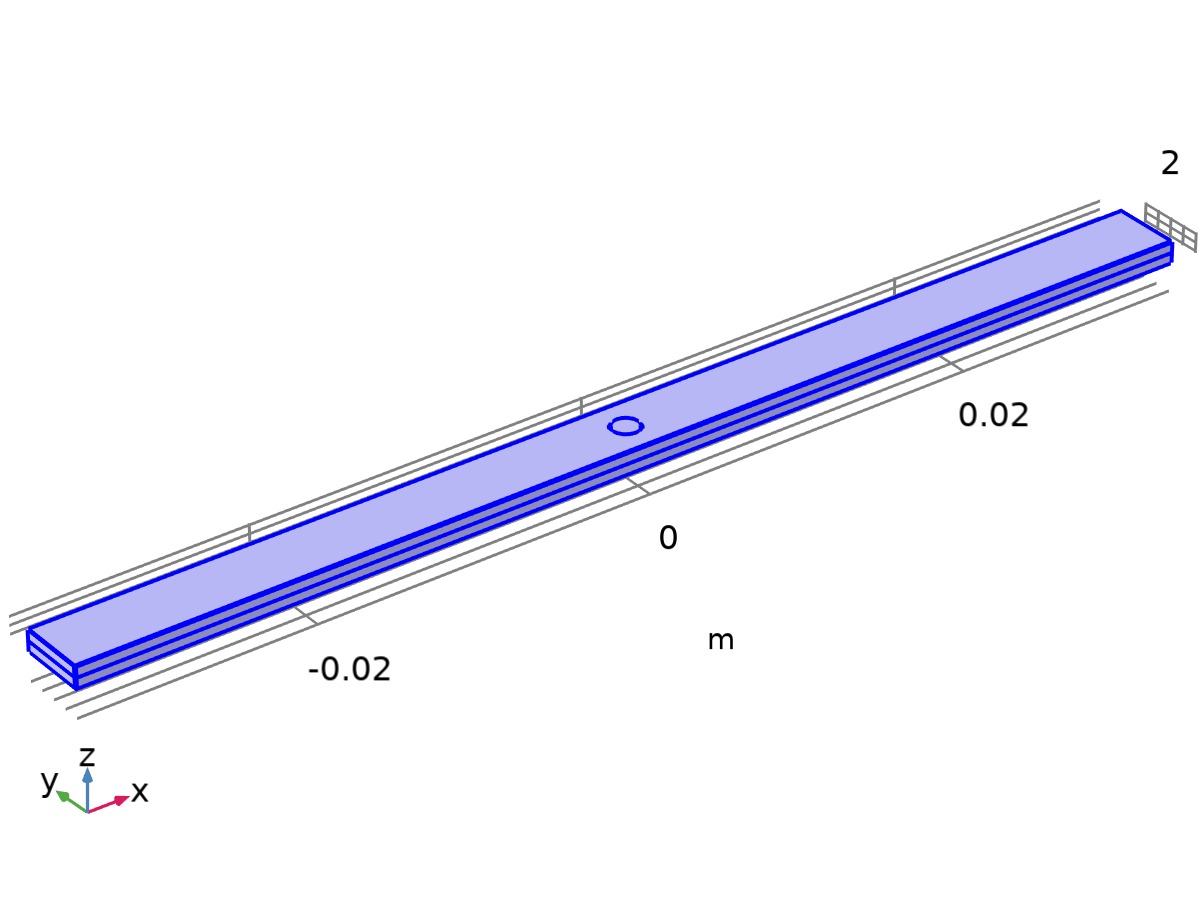


Electric Currents

Selection

| Geometric entity level | Domain |
| --- | --- |
| Selection | Geometry geom1: Dimension 3: All domains |

Equations


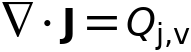


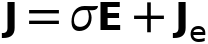


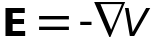


- - 1. Interface Settings

#### Discretization

Settings

| **Description** | **Value** |
| --- | --- |
| Electric potential | Quadratic |

Settings

| **Description** | **Value** |
| --- | --- |
| Equation form | Study controlled |

#### Manual Terminal Sweep Settings

Settings

| **Description** | **Value** | **Unit** |
| --- | --- | --- |
| Use manual terminal sweep | Off |  |
| Reference impedance | 50 | Ω |

- - 1. Variables

| **Name** | **Expression** | **Unit** | **Description** | **Selection** | **Details** |
| --- | --- | --- | --- | --- | --- |
| ec.d | 1 | 1 | Contribution | Domains 1–3 |  |
| ec.I_sXX | (spatial.invF11*(spatial.invF11*ec.I_sxx+spatial.invF21*ec.I_syx+spatial.invF31*ec.I_szx)+spatial.invF21*(spatial.invF11*ec.I_sxy+spatial.invF21*ec.I_syy+spatial.invF31*ec.I_szy)+spatial.invF31*(spatial.invF11*ec.I_sxz+spatial.invF21*ec.I_syz+spatial.invF31*ec.I_szz))*spatial.detF | 1 | Spatial identity matrix, material frame, XX-component | Domains 1–3 |  |
| ec.I_sYX | (spatial.invF11*(spatial.invF12*ec.I_sxx+spatial.invF22*ec.I_syx+spatial.invF32*ec.I_szx)+spatial.invF21*(spatial.invF12*ec.I_sxy+spatial.invF22*ec.I_syy+spatial.invF32*ec.I_szy)+spatial.invF31*(spatial.invF12*ec.I_sxz+spatial.invF22*ec.I_syz+spatial.invF32*ec.I_szz))*spatial.detF | 1 | Spatial identity matrix, material frame, YX-component | Domains 1–3 |  |
| ec.I_sZX | (spatial.invF11*(spatial.invF13*ec.I_sxx+spatial.invF23*ec.I_syx+spatial.invF33*ec.I_szx)+spatial.invF21*(spatial.invF13*ec.I_sxy+spatial.invF23*ec.I_syy+spatial.invF33*ec.I_szy)+spatial.invF31*(spatial.invF13*ec.I_sxz+spatial.invF23*ec.I_syz+spatial.invF33*ec.I_szz))*spatial.detF | 1 | Spatial identity matrix, material frame, ZX-component | Domains 1–3 |  |
| ec.I_sXY | (spatial.invF12*(spatial.invF11*ec.I_sxx+spatial.invF21*ec.I_syx+spatial.invF31*ec.I_szx)+spatial.invF22*(spatial.invF11*ec.I_sxy+spatial.invF21*ec.I_syy+spatial.invF31*ec.I_szy)+spatial.invF32*(spatial.invF11*ec.I_sxz+spatial.invF21*ec.I_syz+spatial.invF31*ec.I_szz))*spatial.detF | 1 | Spatial identity matrix, material frame, XY-component | Domains 1–3 |  |
| ec.I_sYY | (spatial.invF12*(spatial.invF12*ec.I_sxx+spatial.invF22*ec.I_syx+spatial.invF32*ec.I_szx)+spatial.invF22*(spatial.invF12*ec.I_sxy+spatial.invF22*ec.I_syy+spatial.invF32*ec.I_szy)+spatial.invF32*(spatial.invF12*ec.I_sxz+spatial.invF22*ec.I_syz+spatial.invF32*ec.I_szz))*spatial.detF | 1 | Spatial identity matrix, material frame, YY-component | Domains 1–3 |  |
| ec.I_sZY | (spatial.invF12*(spatial.invF13*ec.I_sxx+spatial.invF23*ec.I_syx+spatial.invF33*ec.I_szx)+spatial.invF22*(spatial.invF13*ec.I_sxy+spatial.invF23*ec.I_syy+spatial.invF33*ec.I_szy)+spatial.invF32*(spatial.invF13*ec.I_sxz+spatial.invF23*ec.I_syz+spatial.invF33*ec.I_szz))*spatial.detF | 1 | Spatial identity matrix, material frame, ZY-component | Domains 1–3 |  |
| ec.I_sXZ | (spatial.invF13*(spatial.invF11*ec.I_sxx+spatial.invF21*ec.I_syx+spatial.invF31*ec.I_szx)+spatial.invF23*(spatial.invF11*ec.I_sxy+spatial.invF21*ec.I_syy+spatial.invF31*ec.I_szy)+spatial.invF33*(spatial.invF11*ec.I_sxz+spatial.invF21*ec.I_syz+spatial.invF31*ec.I_szz))*spatial.detF | 1 | Spatial identity matrix, material frame, XZ-component | Domains 1–3 |  |
| ec.I_sYZ | (spatial.invF13*(spatial.invF12*ec.I_sxx+spatial.invF22*ec.I_syx+spatial.invF32*ec.I_szx)+spatial.invF23*(spatial.invF12*ec.I_sxy+spatial.invF22*ec.I_syy+spatial.invF32*ec.I_szy)+spatial.invF33*(spatial.invF12*ec.I_sxz+spatial.invF22*ec.I_syz+spatial.invF32*ec.I_szz))*spatial.detF | 1 | Spatial identity matrix, material frame, YZ-component | Domains 1–3 |  |
| ec.I_sZZ | (spatial.invF13*(spatial.invF13*ec.I_sxx+spatial.invF23*ec.I_syx+spatial.invF33*ec.I_szx)+spatial.invF23*(spatial.invF13*ec.I_sxy+spatial.invF23*ec.I_syy+spatial.invF33*ec.I_szy)+spatial.invF33*(spatial.invF13*ec.I_sxz+spatial.invF23*ec.I_syz+spatial.invF33*ec.I_szz))*spatial.detF | 1 | Spatial identity matrix, material frame, ZZ-component | Domains 1–3 |  |
| ec.I_sxx | 1 | 1 | Spatial identity matrix, xx-component | Domains 1–3 |  |
| ec.I_syx | 0 | 1 | Spatial identity matrix, yx-component | Domains 1–3 |  |
| ec.I_szx | 0 | 1 | Spatial identity matrix, zx-component | Domains 1–3 |  |
| ec.I_sxy | 0 | 1 | Spatial identity matrix, xy-component | Domains 1–3 |  |
| ec.I_syy | 1 | 1 | Spatial identity matrix, yy-component | Domains 1–3 |  |
| ec.I_szy | 0 | 1 | Spatial identity matrix, zy-component | Domains 1–3 |  |
| ec.I_sxz | 0 | 1 | Spatial identity matrix, xz-component | Domains 1–3 |  |
| ec.I_syz | 0 | 1 | Spatial identity matrix, yz-component | Domains 1–3 |  |
| ec.I_szz | 1 | 1 | Spatial identity matrix, zz-component | Domains 1–3 |  |
| ec.nx | nx |  | Normal vector, x-component | Boundaries 6, 9 |  |
| ec.ny | ny |  | Normal vector, y-component | Boundaries 6, 9 |  |
| ec.nz | nz |  | Normal vector, z-component | Boundaries 6, 9 |  |
| ec.nx | dnx |  | Normal vector, x-component | Boundaries 1–5, 7–8, 10–18 |  |
| ec.ny | dny |  | Normal vector, y-component | Boundaries 1–5, 7–8, 10–18 |  |
| ec.nz | dnz |  | Normal vector, z-component | Boundaries 1–5, 7–8, 10–18 |  |
| ec.nmeshx | nxmesh |  | Mesh normal vector, x-component | Boundaries 6, 9 |  |
| ec.nmeshy | nymesh |  | Mesh normal vector, y-component | Boundaries 6, 9 |  |
| ec.nmeshz | nzmesh |  | Mesh normal vector, z-component | Boundaries 6, 9 |  |
| ec.nmeshx | dnxmesh |  | Mesh normal vector, x-component | Boundaries 1–5, 7–8, 10–18 |  |
| ec.nmeshy | dnymesh |  | Mesh normal vector, y-component | Boundaries 1–5, 7–8, 10–18 |  |
| ec.nmeshz | dnzmesh |  | Mesh normal vector, z-component | Boundaries 1–5, 7–8, 10–18 |  |
| ec.unmeshx | unxmesh |  | Mesh normal vector, upside, x-component | Boundaries 1–18 |  |
| ec.unmeshy | unymesh |  | Mesh normal vector, upside, y-component | Boundaries 1–18 |  |
| ec.unmeshz | unzmesh |  | Mesh normal vector, upside, z-component | Boundaries 1–18 |  |
| ec.dnmeshx | dnxmesh |  | Mesh normal vector, downside, x-component | Boundaries 1–18 |  |
| ec.dnmeshy | dnymesh |  | Mesh normal vector, downside, y-component | Boundaries 1–18 |  |
| ec.dnmeshz | dnzmesh |  | Mesh normal vector, downside, z-component | Boundaries 1–18 |  |
| ec.unTx | ec.unTex | Pa | Maxwell upward surface stress tensor, x-component | Boundaries 1–18 |  |
| ec.unTy | ec.unTey | Pa | Maxwell upward surface stress tensor, y-component | Boundaries 1–18 |  |
| ec.unTz | ec.unTez | Pa | Maxwell upward surface stress tensor, z-component | Boundaries 1–18 |  |
| ec.dnTx | ec.dnTex | Pa | Maxwell downward surface stress tensor, x-component | Boundaries 1–18 |  |
| ec.dnTy | ec.dnTey | Pa | Maxwell downward surface stress tensor, y-component | Boundaries 1–18 |  |
| ec.dnTz | ec.dnTez | Pa | Maxwell downward surface stress tensor, z-component | Boundaries 1–18 |  |
| ec.unx | unx |  | Normal vector up direction, x-component | Boundaries 1–18 |  |
| ec.uny | uny |  | Normal vector up direction, y-component | Boundaries 1–18 |  |
| ec.unz | unz |  | Normal vector up direction, z-component | Boundaries 1–18 |  |
| ec.dnx | dnx |  | Normal vector down direction, x-component | Boundaries 1–18 |  |
| ec.dny | dny |  | Normal vector down direction, y-component | Boundaries 1–18 |  |
| ec.dnz | dnz |  | Normal vector down direction, z-component | Boundaries 1–18 |  |
| ec.unTex | -0.5*ec.dnx*(real(up(ec.Dx))*real(up(ec.Ex))+real(up(ec.Dy))*real(up(ec.Ey))+real(up(ec.Dz))*real(up(ec.Ez)))+real(up(ec.Dx))*(real(up(ec.Ex))*ec.dnx+real(up(ec.Ey))*ec.dny+real(up(ec.Ez))*ec.dnz) | Pa | Maxwell upward electric surface stress tensor, x-component | Boundaries 6, 9 |  |
| ec.unTey | -0.5*ec.dny*(real(up(ec.Dx))*real(up(ec.Ex))+real(up(ec.Dy))*real(up(ec.Ey))+real(up(ec.Dz))*real(up(ec.Ez)))+real(up(ec.Dy))*(real(up(ec.Ex))*ec.dnx+real(up(ec.Ey))*ec.dny+real(up(ec.Ez))*ec.dnz) | Pa | Maxwell upward electric surface stress tensor, y-component | Boundaries 6, 9 |  |
| ec.unTez | -0.5*ec.dnz*(real(up(ec.Dx))*real(up(ec.Ex))+real(up(ec.Dy))*real(up(ec.Ey))+real(up(ec.Dz))*real(up(ec.Ez)))+real(up(ec.Dz))*(real(up(ec.Ex))*ec.dnx+real(up(ec.Ey))*ec.dny+real(up(ec.Ez))*ec.dnz) | Pa | Maxwell upward electric surface stress tensor, z-component | Boundaries 6, 9 |  |
| ec.unTex | 0 | Pa | Maxwell upward electric surface stress tensor, x-component | Boundaries 1–5, 7–8, 10–18 |  |
| ec.unTey | 0 | Pa | Maxwell upward electric surface stress tensor, y-component | Boundaries 1–5, 7–8, 10–18 |  |
| ec.unTez | 0 | Pa | Maxwell upward electric surface stress tensor, z-component | Boundaries 1–5, 7–8, 10–18 |  |
| ec.dnTex | -0.5*ec.unx*(real(down(ec.Dx))*real(down(ec.Ex))+real(down(ec.Dy))*real(down(ec.Ey))+real(down(ec.Dz))*real(down(ec.Ez)))+real(down(ec.Dx))*(real(down(ec.Ex))*ec.unx+real(down(ec.Ey))*ec.uny+real(down(ec.Ez))*ec.unz) | Pa | Maxwell downward electric surface stress tensor, x-component | Boundaries 1–18 |  |
| ec.dnTey | -0.5*ec.uny*(real(down(ec.Dx))*real(down(ec.Ex))+real(down(ec.Dy))*real(down(ec.Ey))+real(down(ec.Dz))*real(down(ec.Ez)))+real(down(ec.Dy))*(real(down(ec.Ex))*ec.unx+real(down(ec.Ey))*ec.uny+real(down(ec.Ez))*ec.unz) | Pa | Maxwell downward electric surface stress tensor, y-component | Boundaries 1–18 |  |
| ec.dnTez | -0.5*ec.unz*(real(down(ec.Dx))*real(down(ec.Ex))+real(down(ec.Dy))*real(down(ec.Ey))+real(down(ec.Dz))*real(down(ec.Ez)))+real(down(ec.Dz))*(real(down(ec.Ex))*ec.unx+real(down(ec.Ey))*ec.uny+real(down(ec.Ez))*ec.unz) | Pa | Maxwell downward electric surface stress tensor, z-component | Boundaries 1–18 |  |
| ec.intWe | ec.int_We(ec.d*ec.dWe) | J | Total electric energy | Global | + operation |
| ec.Qh | 0 | W/m³ | Volumetric loss density, electromagnetic | Domains 1–3 |  |
| ec.Qsh | 0 | W/m² | Surface loss density, electromagnetic | Boundaries 1–18 |  |
| ec.Qlh | 0 | W/m | Line loss density, electromagnetic | Edges 1–36 |  |
| ec.CPx | ec.int_CP(x)/ec.int_CP(1) | m | Physics selection center point, spatial frame, x-component | Global |  |
| ec.CPy | ec.int_CP(y)/ec.int_CP(1) | m | Physics selection center point, spatial frame, y-component | Global |  |
| ec.CPz | ec.int_CP(z)/ec.int_CP(1) | m | Physics selection center point, spatial frame, z-component | Global |  |
| ec.R11 | ec.V0_1/ec.I0_1 | Ω | Resistance | Global |  |
| ec.G11 | NaN+NaN*i |  | Conductance | Global |  |
| ec.S11 | NaN+NaN*i |  | S-parameter | Global |  |
| ec.S11dB | 10*log10(realdot(ec.S11,ec.S11)) | dB | S-parameter, dB | Global |  |
| ec.zref | 50[ohm] | Ω | Reference impedance | Global |  |

- - 1. Current Conservation 1


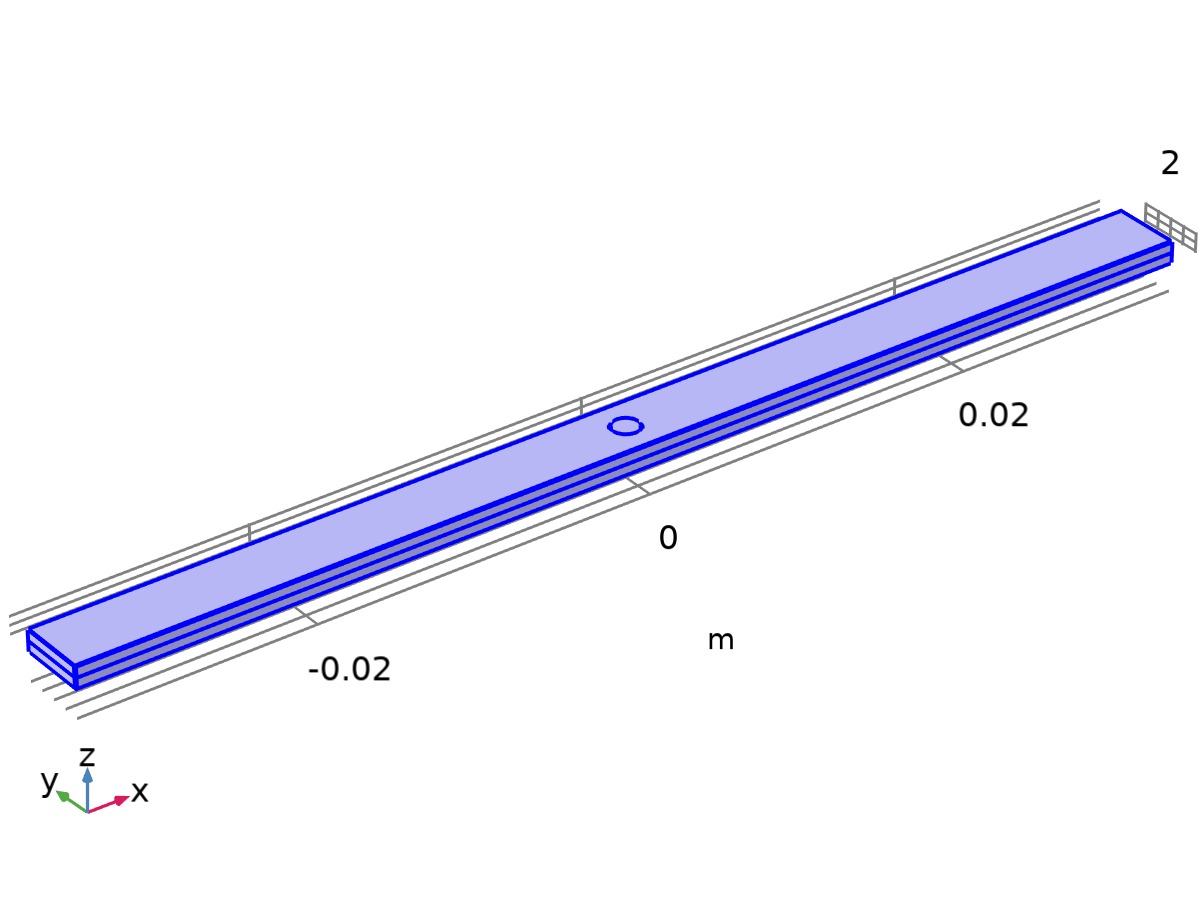


Current Conservation 1

Selection

| Geometric entity level | Domain |
| --- | --- |
| Selection | Geometry geom1: Dimension 3: All domains |

Equations


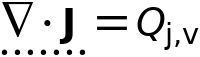


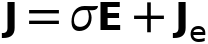


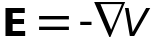


#### Constitutive Relation Jc-E

Settings

| **Description** | **Value** |
| --- | --- |
| Conduction model | Electrical conductivity |
| Electrical conductivity | From material |

#### Constitutive Relation D-E

Settings

| **Description** | **Value** |
| --- | --- |
| Dielectric model | Relative permittivity |
| Relative permittivity | From material |

#### Coordinate System Selection

Settings

| **Description** | **Value** |
| --- | --- |
| Coordinate system | Global coordinate system |

Used products

| COMSOL Multiphysics |
| --- |

Properties from material

| **Property** | **Material** | **Property group** |
| --- | --- | --- |
| Electrical conductivity | Cell Media | Basic |
| Relative permittivity | Cell Media | Basic |
| Electrical conductivity | Cell Layer | Basic |
| Relative permittivity | Cell Layer | Basic |

#### Variables

| **Name** | **Expression** | **Unit** | **Description** | **Selection** | **Details** |
| --- | --- | --- | --- | --- | --- |
| ec.Qh | ec.Qrh | W/m³ | Volumetric loss density, electromagnetic | Domains 1–3 |  |
| ec.Jix | ec.sigmaxx*ec.Ex+ec.sigmaxy*ec.Ey+ec.sigmaxz*ec.Ez | A/m² | Conduction current density, x-component | Domains 1–3 |  |
| ec.Jiy | ec.sigmayx*ec.Ex+ec.sigmayy*ec.Ey+ec.sigmayz*ec.Ez | A/m² | Conduction current density, y-component | Domains 1–3 |  |
| ec.Jiz | ec.sigmazx*ec.Ex+ec.sigmazy*ec.Ey+ec.sigmazz*ec.Ez | A/m² | Conduction current density, z-component | Domains 1–3 |  |
| ec.Jdx | 0 | A/m² | Displacement current density, x-component | Domains 1–3 |  |
| ec.Jdy | 0 | A/m² | Displacement current density, y-component | Domains 1–3 |  |
| ec.Jdz | 0 | A/m² | Displacement current density, z-component | Domains 1–3 |  |
| ec.Jex | 0 | A/m² | External current density, x-component | Domains 1–3 | + operation |
| ec.Jey | 0 | A/m² | External current density, y-component | Domains 1–3 | + operation |
| ec.Jez | 0 | A/m² | External current density, z-component | Domains 1–3 | + operation |
| ec.Jx | ec.Jix+ec.Jdx+ec.Jex | A/m² | Current density, x-component | Domains 1–3 |  |
| ec.Jy | ec.Jiy+ec.Jdy+ec.Jey | A/m² | Current density, y-component | Domains 1–3 |  |
| ec.Jz | ec.Jiz+ec.Jdz+ec.Jez | A/m² | Current density, z-component | Domains 1–3 |  |
| ec.normJ | sqrt(realdot(ec.Jx,ec.Jx)+realdot(ec.Jy,ec.Jy)+realdot(ec.Jz,ec.Jz)) | A/m² | Current density norm | Domains 1–3 |  |
| ec.rhoq | ppr(d(ec.Dx,x)+d(ec.Dy,y)+d(ec.Dz,z)) | C/m³ | Space charge density | Domains 1–3 |  |
| ec.sigmaxx | material.sigma11 | S/m | Electrical conductivity, xx-component | Domains 1–3 | Meta |
| ec.sigmayx | material.sigma21 | S/m | Electrical conductivity, yx-component | Domains 1–3 | Meta |
| ec.sigmazx | material.sigma31 | S/m | Electrical conductivity, zx-component | Domains 1–3 | Meta |
| ec.sigmaxy | material.sigma12 | S/m | Electrical conductivity, xy-component | Domains 1–3 | Meta |
| ec.sigmayy | material.sigma22 | S/m | Electrical conductivity, yy-component | Domains 1–3 | Meta |
| ec.sigmazy | material.sigma32 | S/m | Electrical conductivity, zy-component | Domains 1–3 | Meta |
| ec.sigmaxz | material.sigma13 | S/m | Electrical conductivity, xz-component | Domains 1–3 | Meta |
| ec.sigmayz | material.sigma23 | S/m | Electrical conductivity, yz-component | Domains 1–3 | Meta |
| ec.sigmazz | material.sigma33 | S/m | Electrical conductivity, zz-component | Domains 1–3 | Meta |
| ec.sigma_iso | material.sigma_iso | S/m | Electrical conductivity, isotropic value | Domains 1–3 | Meta |
| ec.epsilonrxx | material.epsilonr11 | 1 | Relative permittivity, xx-component | Domains 1–3 | Meta |
| ec.epsilonryx | material.epsilonr21 | 1 | Relative permittivity, yx-component | Domains 1–3 | Meta |
| ec.epsilonrzx | material.epsilonr31 | 1 | Relative permittivity, zx-component | Domains 1–3 | Meta |
| ec.epsilonrxy | material.epsilonr12 | 1 | Relative permittivity, xy-component | Domains 1–3 | Meta |
| ec.epsilonryy | material.epsilonr22 | 1 | Relative permittivity, yy-component | Domains 1–3 | Meta |
| ec.epsilonrzy | material.epsilonr32 | 1 | Relative permittivity, zy-component | Domains 1–3 | Meta |
| ec.epsilonrxz | material.epsilonr13 | 1 | Relative permittivity, xz-component | Domains 1–3 | Meta |
| ec.epsilonryz | material.epsilonr23 | 1 | Relative permittivity, yz-component | Domains 1–3 | Meta |
| ec.epsilonrzz | material.epsilonr33 | 1 | Relative permittivity, zz-component | Domains 1–3 | Meta |
| ec.epsilonr_iso | material.epsilonr_iso | 1 | Relative permittivity, isotropic value | Domains 1–3 | Meta |
| ec.Dx | epsilon0_const*ec.I_sxx*ec.Ex+epsilon0_const*ec.I_sxy*ec.Ey+epsilon0_const*ec.I_sxz*ec.Ez+ec.Px+ec.Pex+ec.Phx | C/m² | Electric displacement field, x-component | Domains 1–3 |  |
| ec.Dy | epsilon0_const*ec.I_syx*ec.Ex+epsilon0_const*ec.I_syy*ec.Ey+epsilon0_const*ec.I_syz*ec.Ez+ec.Py+ec.Pey+ec.Phy | C/m² | Electric displacement field, y-component | Domains 1–3 |  |
| ec.Dz | epsilon0_const*ec.I_szx*ec.Ex+epsilon0_const*ec.I_szy*ec.Ey+epsilon0_const*ec.I_szz*ec.Ez+ec.Pz+ec.Pez+ec.Phz | C/m² | Electric displacement field, z-component | Domains 1–3 |  |
| ec.Px | epsilon0_const*(ec.chixx*ec.Ex+ec.chixy*ec.Ey+ec.chixz*ec.Ez) | C/m² | Polarization, x-component | Domains 1–3 |  |
| ec.Py | epsilon0_const*(ec.chiyx*ec.Ex+ec.chiyy*ec.Ey+ec.chiyz*ec.Ez) | C/m² | Polarization, y-component | Domains 1–3 |  |
| ec.Pz | epsilon0_const*(ec.chizx*ec.Ex+ec.chizy*ec.Ey+ec.chizz*ec.Ez) | C/m² | Polarization, z-component | Domains 1–3 |  |
| ec.normD | sqrt(realdot(ec.Dx,ec.Dx)+realdot(ec.Dy,ec.Dy)+realdot(ec.Dz,ec.Dz)) | C/m² | Electric displacement field norm | Domains 1–3 |  |
| ec.normP | sqrt(realdot(ec.Px,ec.Px)+realdot(ec.Py,ec.Py)+realdot(ec.Pz,ec.Pz)) | C/m² | Polarization norm | Domains 1–3 |  |
| ec.Pex | 0 | C/m² | Polarization contribution, x-component | Domains 1–3 | + operation |
| ec.Pey | 0 | C/m² | Polarization contribution, y-component | Domains 1–3 | + operation |
| ec.Pez | 0 | C/m² | Polarization contribution, z-component | Domains 1–3 | + operation |
| ec.Phx | 0 | C/m² | Polarization contribution, x-component | Domains 1–3 | + operation |
| ec.Phy | 0 | C/m² | Polarization contribution, y-component | Domains 1–3 | + operation |
| ec.Phz | 0 | C/m² | Polarization contribution, z-component | Domains 1–3 | + operation |
| ec.chixx | -1+ec.epsilonrxx | 1 | Electric susceptibility, xx-component | Domains 1–3 |  |
| ec.chiyx | ec.epsilonryx | 1 | Electric susceptibility, yx-component | Domains 1–3 |  |
| ec.chizx | ec.epsilonrzx | 1 | Electric susceptibility, zx-component | Domains 1–3 |  |
| ec.chixy | ec.epsilonrxy | 1 | Electric susceptibility, xy-component | Domains 1–3 |  |
| ec.chiyy | -1+ec.epsilonryy | 1 | Electric susceptibility, yy-component | Domains 1–3 |  |
| ec.chizy | ec.epsilonrzy | 1 | Electric susceptibility, zy-component | Domains 1–3 |  |
| ec.chixz | ec.epsilonrxz | 1 | Electric susceptibility, xz-component | Domains 1–3 |  |
| ec.chiyz | ec.epsilonryz | 1 | Electric susceptibility, yz-component | Domains 1–3 |  |
| ec.chizz | -1+ec.epsilonrzz | 1 | Electric susceptibility, zz-component | Domains 1–3 |  |
| ec.Ex | -Vx | V/m | Electric field, x-component | Domains 1–3 |  |
| ec.Ey | -Vy | V/m | Electric field, y-component | Domains 1–3 |  |
| ec.Ez | -Vz | V/m | Electric field, z-component | Domains 1–3 |  |
| ec.tEx | -VTx | V/m | Tangential electric field, x-component | Boundaries 1–18 |  |
| ec.tEy | -VTy | V/m | Tangential electric field, y-component | Boundaries 1–18 |  |
| ec.tEz | -VTz | V/m | Tangential electric field, z-component | Boundaries 1–18 |  |
| ec.normE | sqrt(realdot(ec.Ex,ec.Ex)+realdot(ec.Ey,ec.Ey)+realdot(ec.Ez,ec.Ez)) | V/m | Electric field norm | Domains 1–3 |  |
| ec.Qrh | ec.Jx*ec.Ex+ec.Jy*ec.Ey+ec.Jz*ec.Ez | W/m³ | Volumetric loss density, electric | Domains 1–3 | + operation |
| ec.W | ec.We | J/m³ | Energy density | Domains 1–3 | + operation |
| ec.dWe | ec.We | J/m³ | Integrand for total electric energy | Domains 1–3 | Meta |
| ec.We | 0.5*epsilon0_const*(((ec.I_sxx+ec.chixx)*ec.Ex+(ec.I_sxy+ec.chixy)*ec.Ey+(ec.I_sxz+ec.chixz)*ec.Ez)*ec.Ex+((ec.I_syx+ec.chiyx)*ec.Ex+(ec.I_syy+ec.chiyy)*ec.Ey+(ec.I_syz+ec.chiyz)*ec.Ez)*ec.Ey+((ec.I_szx+ec.chizx)*ec.Ex+(ec.I_szy+ec.chizy)*ec.Ey+(ec.I_szz+ec.chizz)*ec.Ez)*ec.Ez) | J/m³ | Electric energy density | Domains 1–3 |  |
| ec.rhoqs | ec.dnx*(up(ec.Dx)-down(ec.Dx))+ec.dny*(up(ec.Dy)-down(ec.Dy))+ec.dnz*(up(ec.Dz)-down(ec.Dz)) | C/m² | Surface charge density | Boundaries 6, 9 |  |
| ec.rhoqs | -ec.dnx*down(ec.Dx)-ec.dny*down(ec.Dy)-ec.dnz*down(ec.Dz) | C/m² | Surface charge density | Boundaries 1–5, 7–8, 10–18 |  |

#### Shape functions

| **Name** | **Shape function** | **Unit** | **Description** | **Shape frame** | **Selection** |
| --- | --- | --- | --- | --- | --- |
| V | Lagrange (Quadratic) | V | Electric potential | Spatial | Domains 1–3 |
| V | Lagrange (Quadratic) | V | Electric potential | Material | Domains 1–3 |
| V | Lagrange (Quadratic) | V | Electric potential | Geometry | Domains 1–3 |
| V | Lagrange (Quadratic) | V | Electric potential | Mesh | Domains 1–3 |

#### Weak Expressions

| **Weak expression** | **Integration order** | **Integration frame** | **Selection** |
| --- | --- | --- | --- |
| (ec.Jx*test(Vx)+ec.Jy*test(Vy)+ec.Jz*test(Vz))*ec.d | 4 | Spatial | Domains 1–3 |

- - 1. Electric Insulation 1


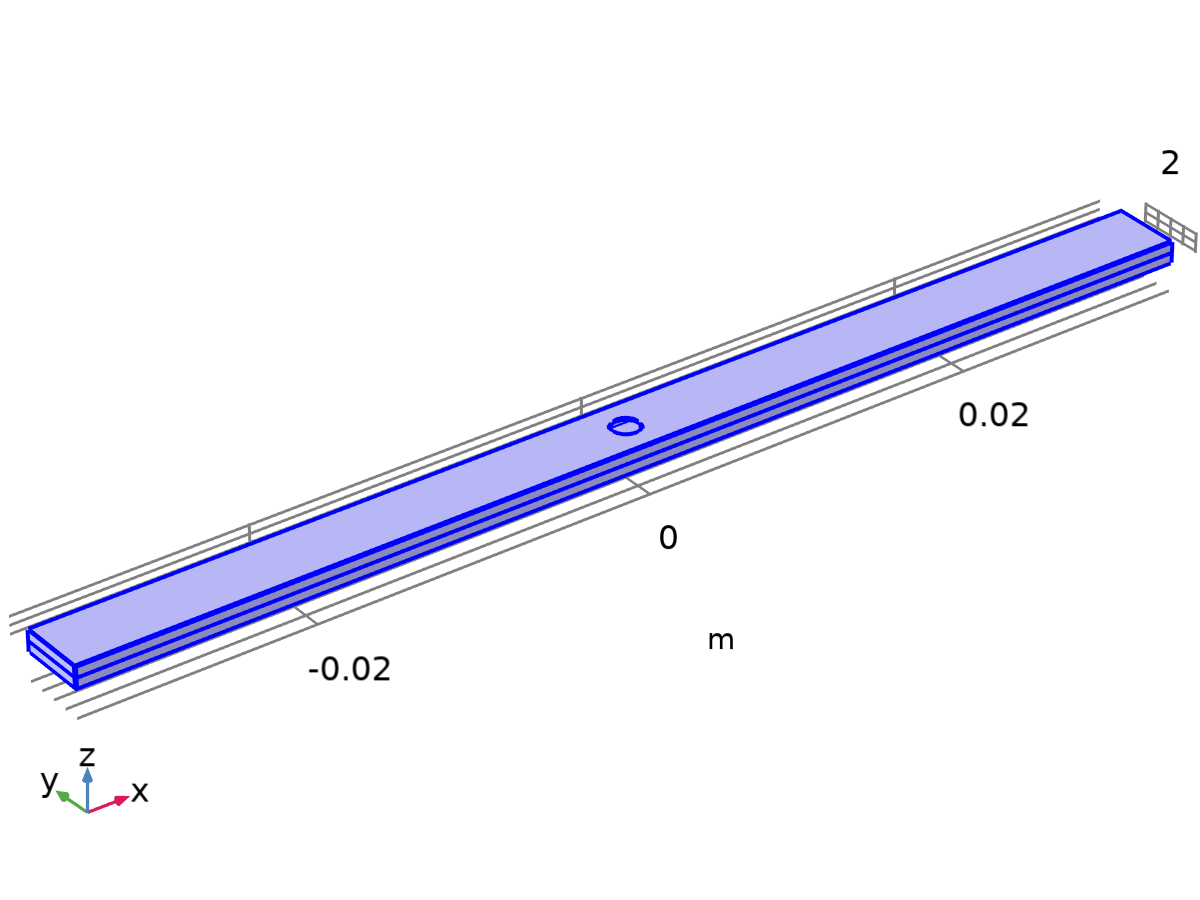


Electric Insulation 1

Selection

| Geometric entity level | Boundary |
| --- | --- |
| Selection | Geometry geom1: Dimension 2: All boundaries |

Equations


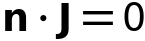


Used products

| COMSOL Multiphysics |
| --- |

#### Variables

| **Name** | **Expression** | **Unit** | **Description** | **Selection** | **Details** |
| --- | --- | --- | --- | --- | --- |
| ec.nJ | 0 | A/m² | Normal current density | Boundaries 1–5, 7–8, 10–13, 16–18 | + operation |

#### Shape functions

| **Name** | **Shape function** | **Unit** | **Description** | **Shape frame** | **Selection** | **Details** |
| --- | --- | --- | --- | --- | --- | --- |
| V | Lagrange (Quadratic) | V | Electric potential | Spatial | No boundaries | Slit |
| V | Lagrange (Quadratic) | V | Electric potential | Material | No boundaries | Slit |
| V | Lagrange (Quadratic) | V | Electric potential | Geometry | No boundaries | Slit |
| V | Lagrange (Quadratic) | V | Electric potential | Mesh | No boundaries | Slit |

- - 1. Initial Values 1


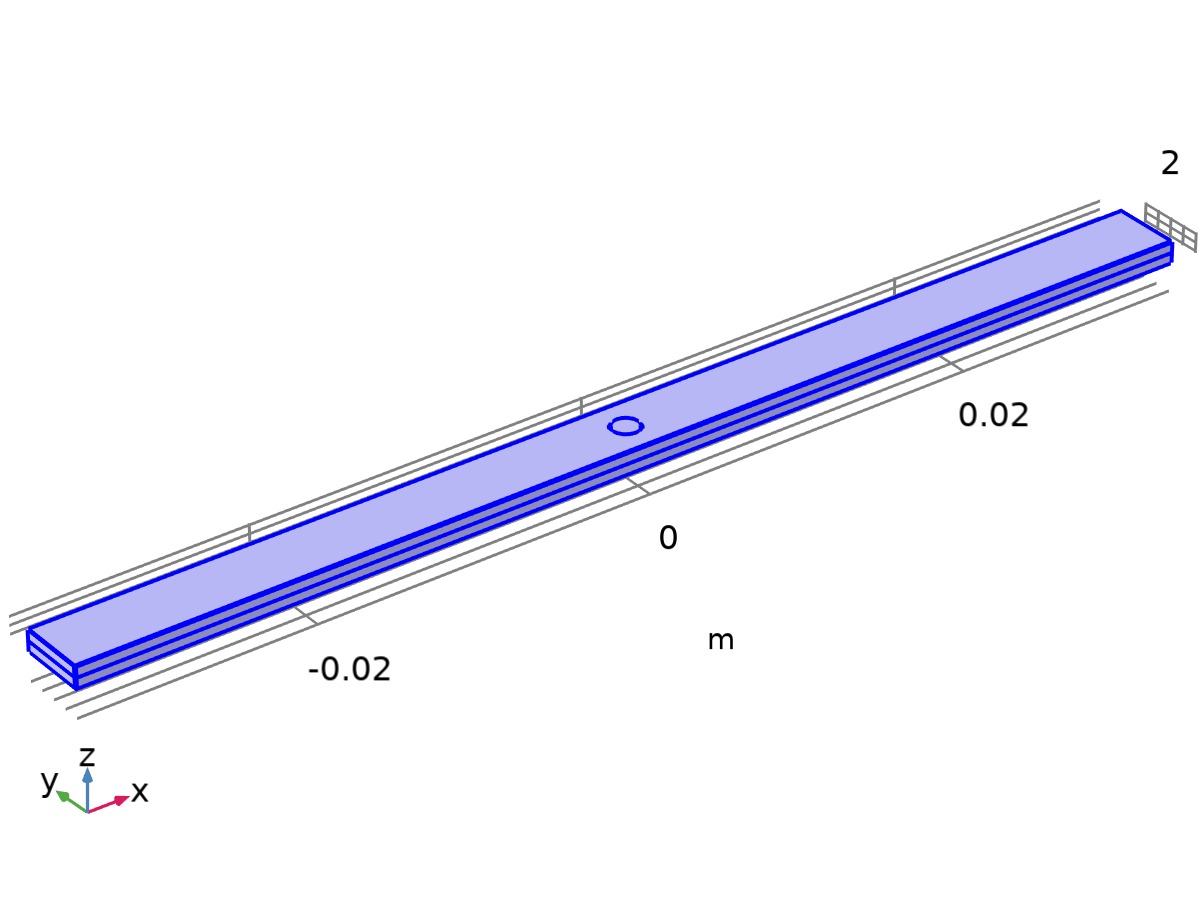


Initial Values 1

Selection

| Geometric entity level | Domain |
| --- | --- |
| Selection | Geometry geom1: Dimension 3: All domains |

Settings

| **Description** | **Value** | **Unit** |
| --- | --- | --- |
| Electric potential | 0 | V |

Used products

| COMSOL Multiphysics |
| --- |

- - 1. Terminal 1


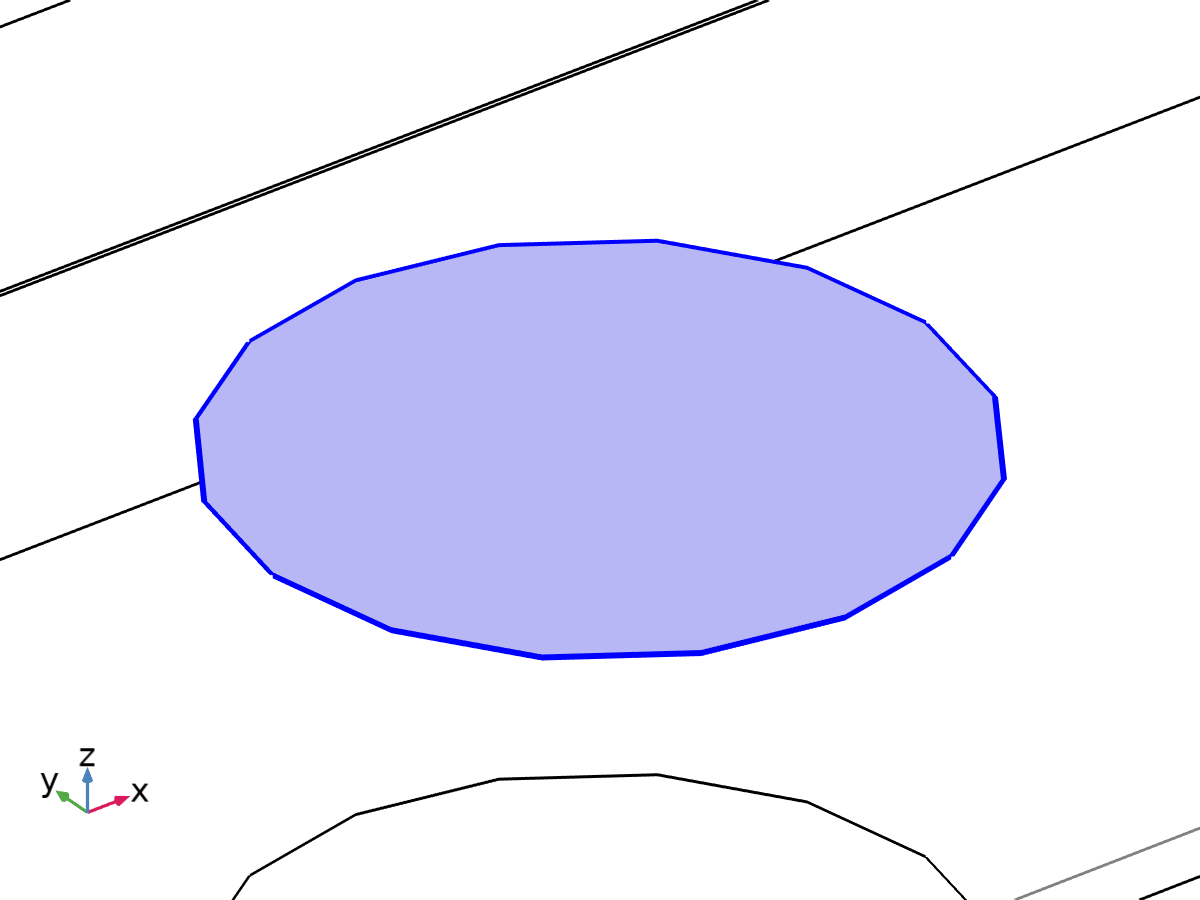


Terminal 1

Selection

| Geometric entity level | Boundary |
| --- | --- |
| Selection | Geometry geom1: Dimension 2: Boundary 15 |

Equations


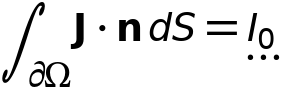


#### Terminal

Settings

| **Description** | **Value** | **Unit** |
| --- | --- | --- |
| Terminal name | 1 |  |
| Terminal type | Current |  |
| Current | 1E-6 | A |

#### Variables

| **Name** | **Expression** | **Unit** | **Description** | **Selection** | **Details** |
| --- | --- | --- | --- | --- | --- |
| ec.nJ | ec.unx*down(ec.Jx)+ec.uny*down(ec.Jy)+ec.unz*down(ec.Jz) | A/m² | Normal current density | Boundary 15 | + operation |
| ec.I0 | 1[uA] | A | Current | Boundary 15 |  |
| ec.term1.Vinit | 0[V] | V | Initial value for voltage | Global |  |
| ec.I0_1 | ec.term1.int(ec.I0)/ec.term1.int(1) | A | Terminal current | Global |  |
| ec.V0_1 | ec.term1.V0_ode | V | Terminal voltage | Global |  |

#### Shape functions

| **Name** | **Shape function** | **Unit** | **Description** | **Shape frame** | **Selection** |
| --- | --- | --- | --- | --- | --- |
| ec.term1.V0_ode | ODE | V | Terminal voltage |  | Global |

#### Weak Expressions

| **Weak expression** | **Integration order** | **Integration frame** | **Selection** |
| --- | --- | --- | --- |
| ec.I0_1*test(ec.term1.V0_ode) | 4 |  | Global |

#### Constraints

| **Constraint** | **Constraint force** | **Shape function** | **Selection** | **Details** |
| --- | --- | --- | --- | --- |
| ec.term1.V0_ode-V | test(ec.term1.V0_ode-V) | Lagrange (Quadratic) | Boundary 15 | Elemental |

- - 1. Ground 1


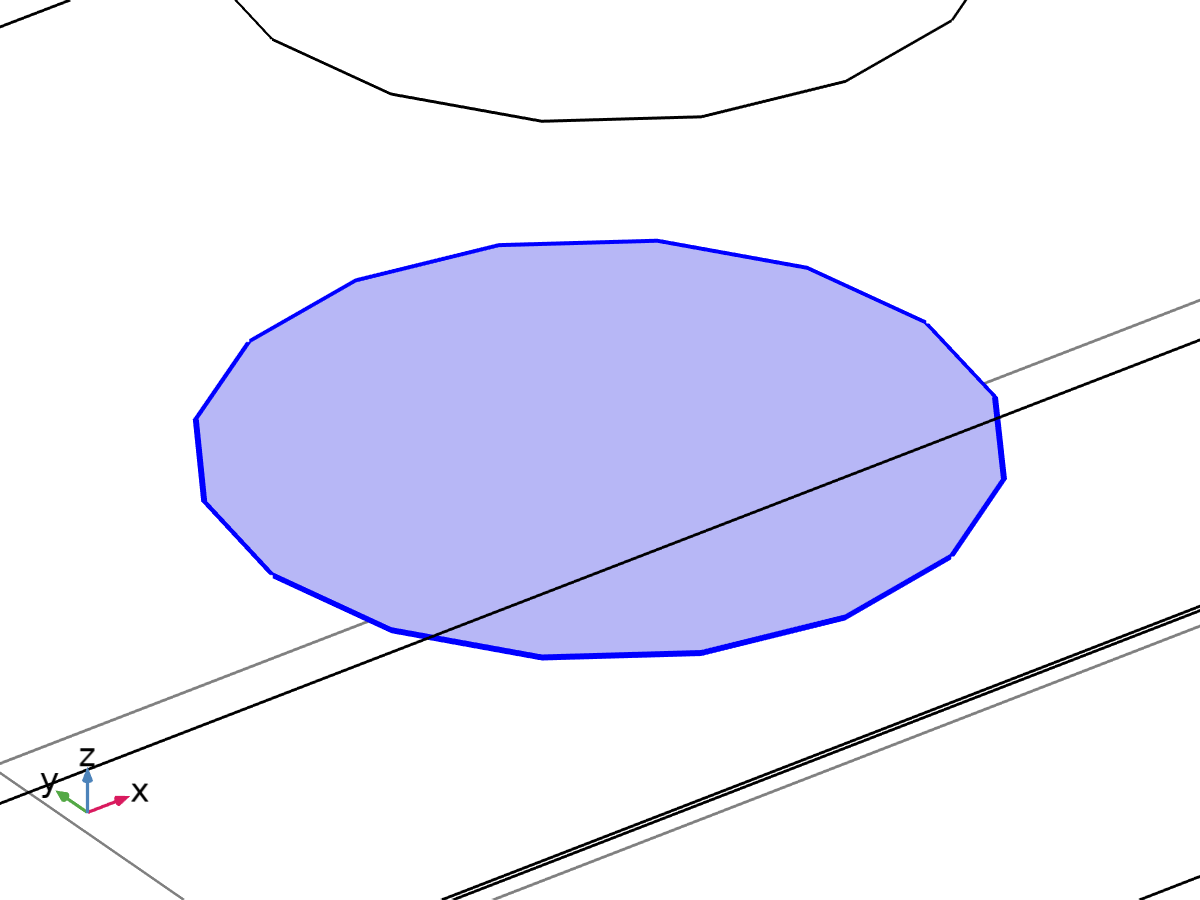


Ground 1

Selection

| Geometric entity level | Boundary |
| --- | --- |
| Selection | Geometry geom1: Dimension 2: Boundary 14 |

Equations


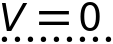


Used products

| COMSOL Multiphysics |
| --- |

#### Variables

| **Name** | **Expression** | **Unit** | **Description** | **Selection** | **Details** |
| --- | --- | --- | --- | --- | --- |
| ec.nJ | ec.unx*down(ec.Jx)+ec.uny*down(ec.Jy)+ec.unz*down(ec.Jz) | A/m² | Normal current density | Boundary 14 | + operation |
| ec.V0 | 0 | V | Electric potential | Boundary 14 |  |

#### Constraints

| **Constraint** | **Constraint force** | **Shape function** | **Selection** | **Details** |
| --- | --- | --- | --- | --- |
| ec.V0-V | test(ec.V0-V) | Lagrange (Quadratic) | Boundary 14 | Elemental |

- 1. Mesh 1


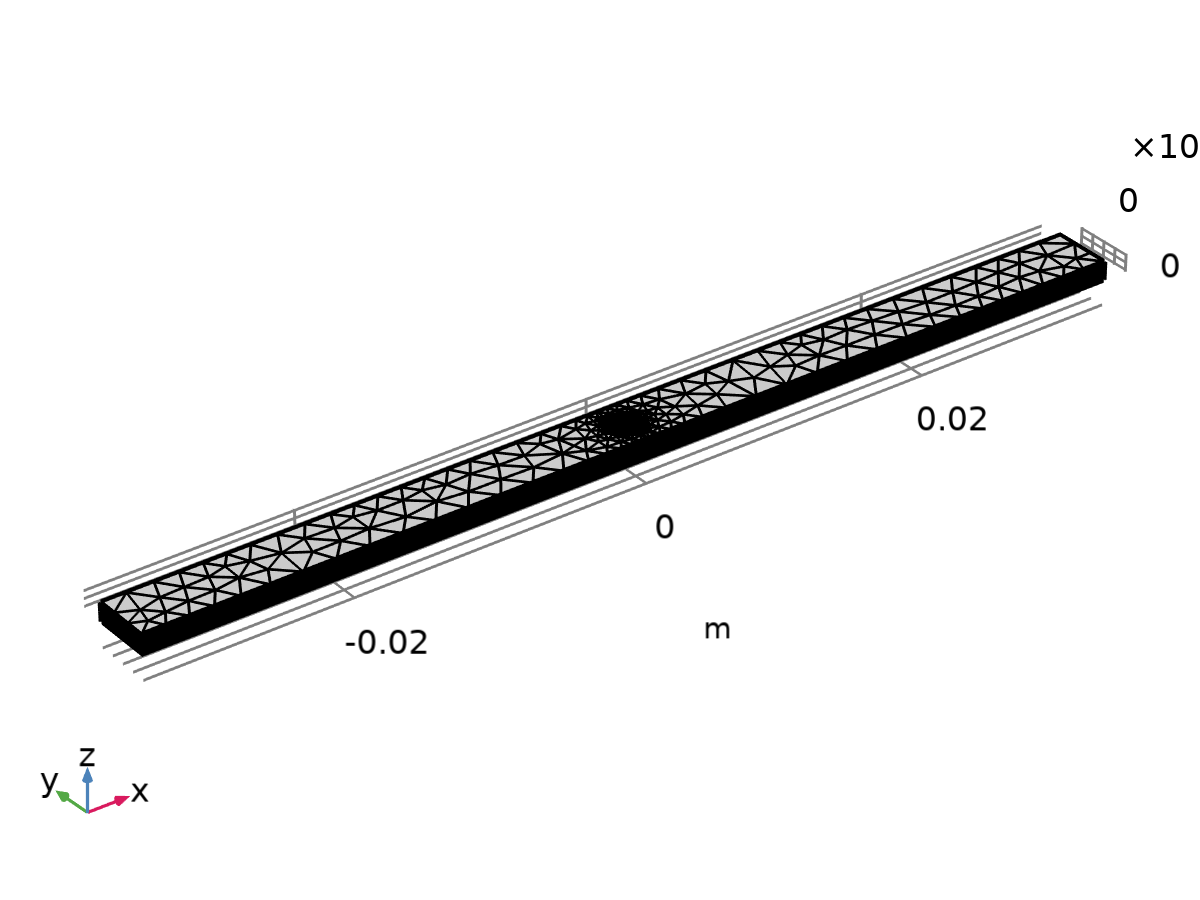


Mesh 1

Mesh statistics

| **Description** | **Value** |
| --- | --- |
| Status | Complete mesh |
| Mesh vertices | 176196 |
| Tetrahedra | 1017728 |
| Triangles | 118174 |
| Edge elements | 2735 |
| Vertex elements | 24 |
| Number of elements | 1017728 |
| Minimum element quality | 0.02332 |
| Average element quality | 0.1198 |
| Element volume ratio | 1.5553E-5 |
| Mesh volume | 3.299E-7 m³ |

- - 1. Size (size)

Settings

| **Description** | **Value** |
| --- | --- |
| Maximum element size | 0.00236 |
| Minimum element size | 1.01E-4 |
| Curvature factor | 0.3 |
| Resolution of narrow regions | 0.85 |
| Maximum element growth rate | 1.35 |
| Predefined size | Extra fine |

- - 1. Free Tetrahedral 1 (ftet1)

Selection

| Geometric entity level | Domain |
| --- | --- |
| Selection | Remaining |


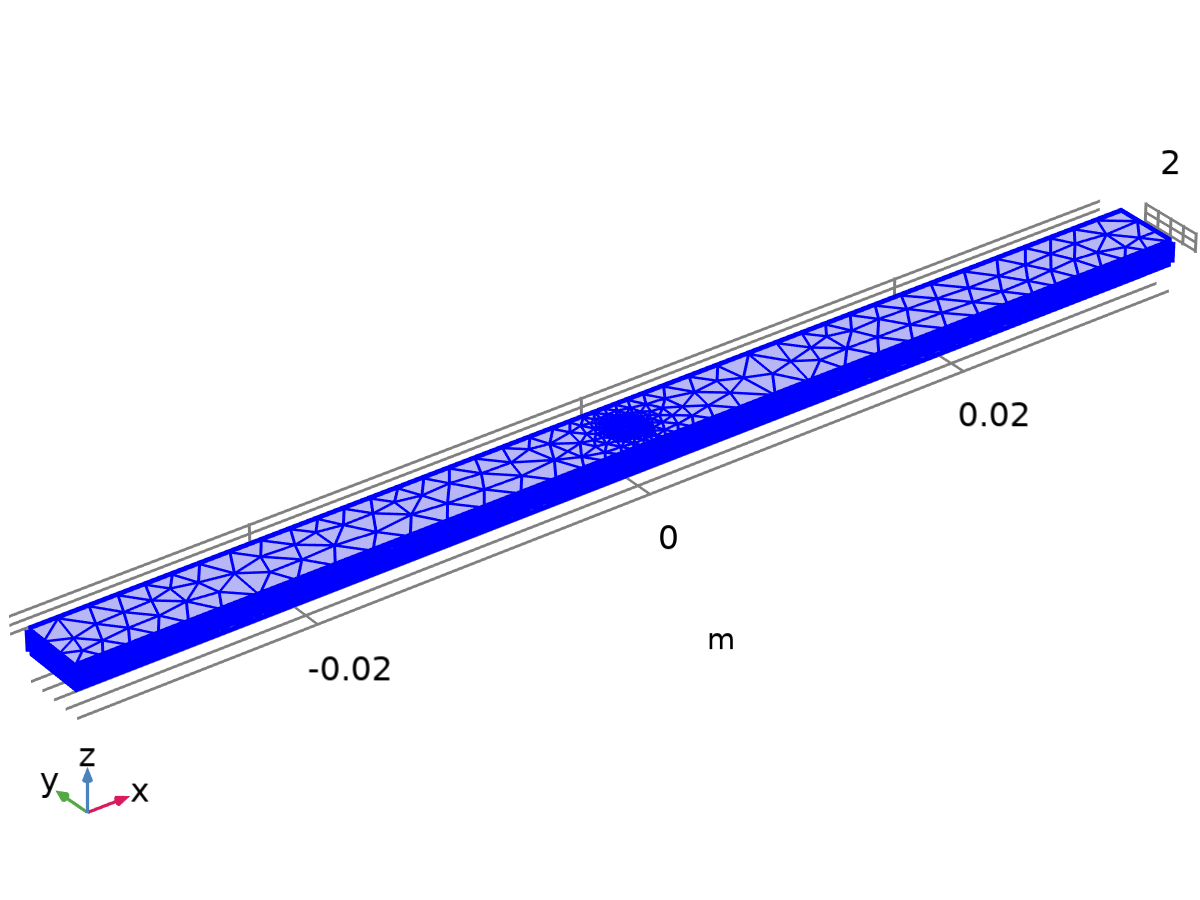


Free Tetrahedral 1

Settings

| **Description** | **Value** |
| --- | --- |
| z-direction scale | 10 |
| Avoid inverted curved elements | On |
| Last build time | 14 |
| Built with | COMSOL 6.1.0.252 (win64)\|2023 - 06 - 16T09:06:38.044822400 |

1. Study 1

Computation information

| Computation time | 17 min 24 s |
| --- | --- |

- 1. Parametric Sweep

| **Parameter name** | **Parameter value list** | **Parameter unit** |
| --- | --- | --- |
| barrier_g | 10^{range(log10(7.5),-(1/4),log10(7.5e-5))} | S/m |

Study settings

| **Description** | **Value** |
| --- | --- |
| Sweep type | Specified combinations |
| Parameter name | barrier_g |
| Unit | S/m |

Parameters

| **Parameter name** | **Parameter value list** | **Parameter unit** |
| --- | --- | --- |
| barrier_g | 10^{range(log10(7.5),-(1/4),log10(7.5e-5))} | S/m |

- 1. Stationary

Study settings

| **Description** | **Value** |
| --- | --- |
| Include geometric nonlinearity | Off |

Physics and variables selection

| **Physics interface** | **Discretization** |
| --- | --- |
| Electric Currents (ec) | physics |

Mesh selection

| **Geometry** | **Mesh** |
| --- | --- |
| Geometry 1 (geom1) | mesh1 |

- 1. Solver Configurations
     1. Solution 1

#### Compile Equations: Stationary (st1)

Study and step

| **Description** | **Value** |
| --- | --- |
| Use study | [Study 1](#cs2545333) |
| Use study step | Stationary |

Log

<---- Compile Equations: Stationary in Study 1/Solution 1 (sol1) ---------------

Started at Jun 16, 2023, 9:38:44 AM.

Geometry shape function: Quadratic Lagrange

Running on Intel64 Family 6 Model 158 Stepping 9, GenuineIntel.

Using 1 socket with 4 cores in total on BJORN_ULTRALJUD.

Available memory: 16.24 GB.

Time: 6 s.

Physical memory: 1.86 GB

Virtual memory: 2.08 GB

Ended at Jun 16, 2023, 9:38:50 AM.

----- Compile Equations: Stationary in Study 1/Solution 1 (sol1) -------------->

#### Dependent Variables 1 (v1)

General

| **Description** | **Value** |
| --- | --- |
| Defined by study step | [Stationary](#cs7679031) |

Initial value calculation constants

| **Constant name** | **Initial value source** |
| --- | --- |
| barrier_g | 10^{range(log10(7.5),-(1/4),log10(7.5e-5))}[S/m] |

Log

<---- Dependent Variables 1 in Study 1/Solution 1 (sol1) -----------------------

Started at Jun 16, 2023, 9:38:50 AM.

Solution time: 1 s.

Physical memory: 1.77 GB

Virtual memory: 1.96 GB

Ended at Jun 16, 2023, 9:38:50 AM.

----- Dependent Variables 1 in Study 1/Solution 1 (sol1) ---------------------->

##### Electric potential (comp1.V) (comp1_V)

General

| **Description** | **Value** |
| --- | --- |
| Field components | comp1.V |

##### Terminal voltage (comp1.ec.term1.V0_ode) (comp1_ec_term1_V0_ode)

General

| **Description** | **Value** |
| --- | --- |
| State components | comp1.ec.term1.V0_ode |

#### Stationary Solver 1 (s1)

General

| **Description** | **Value** |
| --- | --- |
| Defined by study step | [Stationary](#cs7679031) |

Results while solving

| **Description** | **Value** |
| --- | --- |
| Probes | None |

Log

<---- Stationary Solver 1 in Study 1/Solution 1 (sol1) -------------------------

Started at Jun 16, 2023, 9:38:51 AM.

Parametric solver

Linear solver

Number of degrees of freedom solved for: 1383279.

Parameter barrier_g = 7.5.

Symmetric matrices found.

Scales for dependent variables:

Electric potential (comp1.V): 1

Terminal voltage (comp1.ec.term1.V0_ode): 0.041

Sparse null-space function used.

Iter      SolEst     Damping    Stepsize #Res #Jac #Sol LinIt   LinErr   LinRes

   1           1   1.0000000           1    1    1    1    72   0.0007  1.2e-05

Parameter barrier_g = 4.21756.

Iter      SolEst     Damping    Stepsize #Res #Jac #Sol LinIt   LinErr   LinRes

   1           1   1.0000000           1    2    2    2   142  0.00085  1.5e-05

Parameter barrier_g = 2.37171.

Iter      SolEst     Damping    Stepsize #Res #Jac #Sol LinIt   LinErr   LinRes

   1           1   1.0000000           1    3    3    3   212  0.00081  1.3e-05

Parameter barrier_g = 1.33371.

Iter      SolEst     Damping    Stepsize #Res #Jac #Sol LinIt   LinErr   LinRes

   1           1   1.0000000           1    4    4    4   283  0.00093  1.8e-05

Parameter barrier_g = 0.75.

Iter      SolEst     Damping    Stepsize #Res #Jac #Sol LinIt   LinErr   LinRes

   1           1   1.0000000           1    5    5    5   350  0.00089  1.6e-05

Parameter barrier_g = 0.421756.

Iter      SolEst     Damping    Stepsize #Res #Jac #Sol LinIt   LinErr   LinRes

   1           1   1.0000000           1    6    6    6   417  0.00074  1.5e-05

Parameter barrier_g = 0.237171.

Iter      SolEst     Damping    Stepsize #Res #Jac #Sol LinIt   LinErr   LinRes

   1           1   1.0000000           1    7    7    7   482   0.0008  1.7e-05

Parameter barrier_g = 0.133371.

Iter      SolEst     Damping    Stepsize #Res #Jac #Sol LinIt   LinErr   LinRes

   1           1   1.0000000           1    8    8    8   549  0.00071  1.5e-05

Parameter barrier_g = 0.075.

Iter      SolEst     Damping    Stepsize #Res #Jac #Sol LinIt   LinErr   LinRes

   1           1   1.0000000           1    9    9    9   615  0.00079  1.4e-05

Parameter barrier_g = 0.0421756.

Iter      SolEst     Damping    Stepsize #Res #Jac #Sol LinIt   LinErr   LinRes

   1           1   1.0000000           1   10   10   10   680   0.0007  1.6e-05

Parameter barrier_g = 0.0237171.

Iter      SolEst     Damping    Stepsize #Res #Jac #Sol LinIt   LinErr   LinRes

   1           1   1.0000000           1   11   11   11   747  0.00073  1.4e-05

Parameter barrier_g = 0.0133371.

Iter      SolEst     Damping    Stepsize #Res #Jac #Sol LinIt   LinErr   LinRes

   1           1   1.0000000           1   12   12   12   812  0.00093  2.3e-05

Parameter barrier_g = 0.0075.

Iter      SolEst     Damping    Stepsize #Res #Jac #Sol LinIt   LinErr   LinRes

   1           1   1.0000000           1   13   13   13   878  0.00072  1.7e-05

Parameter barrier_g = 0.00421756.

Iter      SolEst     Damping    Stepsize #Res #Jac #Sol LinIt   LinErr   LinRes

   1           1   1.0000000           1   14   14   14   948  0.00064  1.4e-05

Parameter barrier_g = 0.00237171.

Iter      SolEst     Damping    Stepsize #Res #Jac #Sol LinIt   LinErr   LinRes

   1           1   1.0000000           1   15   15   15  1018  0.00084  1.8e-05

Parameter barrier_g = 0.00133371.

Iter      SolEst     Damping    Stepsize #Res #Jac #Sol LinIt   LinErr   LinRes

   1           1   1.0000000           1   16   16   16  1091  0.00078  1.6e-05

Parameter barrier_g = 0.00075.

Iter      SolEst     Damping    Stepsize #Res #Jac #Sol LinIt   LinErr   LinRes

   1           1   1.0000000           1   17   17   17  1172  0.00084  1.4e-05

Parameter barrier_g = 0.000421756.

Iter      SolEst     Damping    Stepsize #Res #Jac #Sol LinIt   LinErr   LinRes

   1           1   1.0000000           1   18   18   18  1258  0.00091  1.6e-05

Parameter barrier_g = 0.000237171.

Iter      SolEst     Damping    Stepsize #Res #Jac #Sol LinIt   LinErr   LinRes

   1           1   1.0000000           1   19   19   19  1357   0.0008  1.5e-05

Parameter barrier_g = 0.000133371.

Iter      SolEst     Damping    Stepsize #Res #Jac #Sol LinIt   LinErr   LinRes

   1           1   1.0000000           1   20   20   20  1459  0.00098  1.5e-05

Parameter barrier_g = 7.5e-05.

Iter      SolEst     Damping    Stepsize #Res #Jac #Sol LinIt   LinErr   LinRes

   1           1   1.0000000           1   21   21   21  1566  0.00083  2.1e-05

Solution time: 1037 s. (17 minutes, 17 seconds)

Physical memory: 2.84 GB

Virtual memory: 3 GB

Ended at Jun 16, 2023, 9:56:08 AM.

----- Stationary Solver 1 in Study 1/Solution 1 (sol1) ------------------------>

##### Parametric 1 (p1)

General

| **Description** | **Value** |
| --- | --- |
| Defined by study step | [Parametric Sweep](#cs5685825) |
| Run continuation for | No parameter |

Parameters

| **Parameter name** | **Parameter value list** | **Parameter unit** |
| --- | --- | --- |
| barrier_g | 10^{range(log10(7.5),-(1/4),log10(7.5e-5))} | S/m |

##### Fully Coupled 1 (fc1)

General

| **Description** | **Value** |
| --- | --- |
| Linear solver | [Iterative 1](#cs8565931) |

##### Iterative 1 (i1)

General

| **Description** | **Value** |
| --- | --- |
| Solver | Conjugate gradients |

###### Multigrid 1 (mg1)

General

| **Description** | **Value** |
| --- | --- |
| Solver | Algebraic multigrid |
| Coarsening method | Classical |

1. Results
   1. Datasets
      1. Study 1/Solution 1

Solution

| **Description** | **Value** |
| --- | --- |
| Solution | [Solution 1](#cs5127424) |
| Component | Component 1 (comp1) |


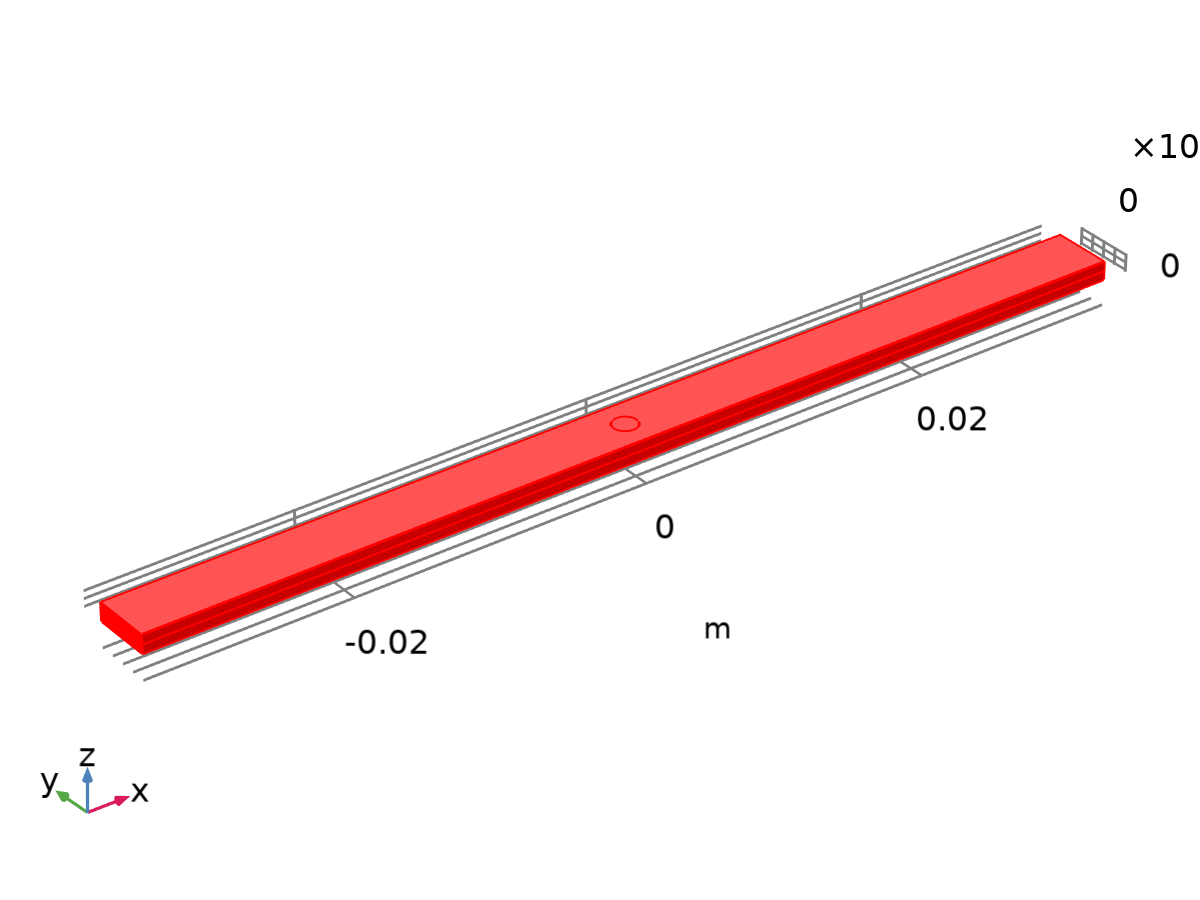


Dataset: Study 1/Solution 1

- 1. Derived Values
     1. Global Evaluation 1

Output

| Evaluated in | Table 1 |
| --- | --- |

Data

| **Description** | **Value** |
| --- | --- |
| Dataset | [Study 1/Solution 1](#cs6653902) |

Expressions

| **Expression** | **Unit** | **Description** |
| --- | --- | --- |
| barrier_h/barrier_g | Ω*cm^2 | True TEER |
| ec.R11 | Ω | Resistance |

- 1. Plot Groups
     1. Electric Potential (ec)


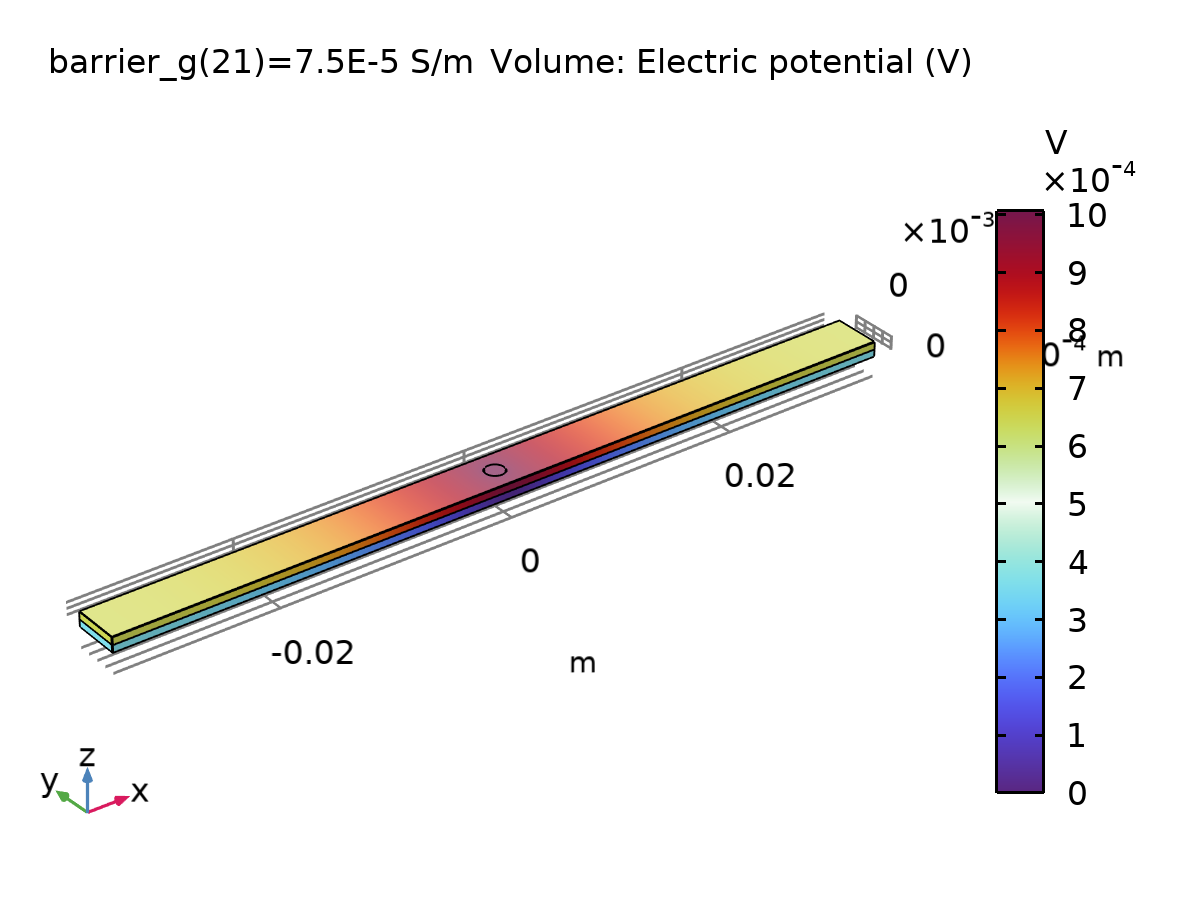


Volume: Electric potential (V)

- - 1. Electric Field Norm (ec)


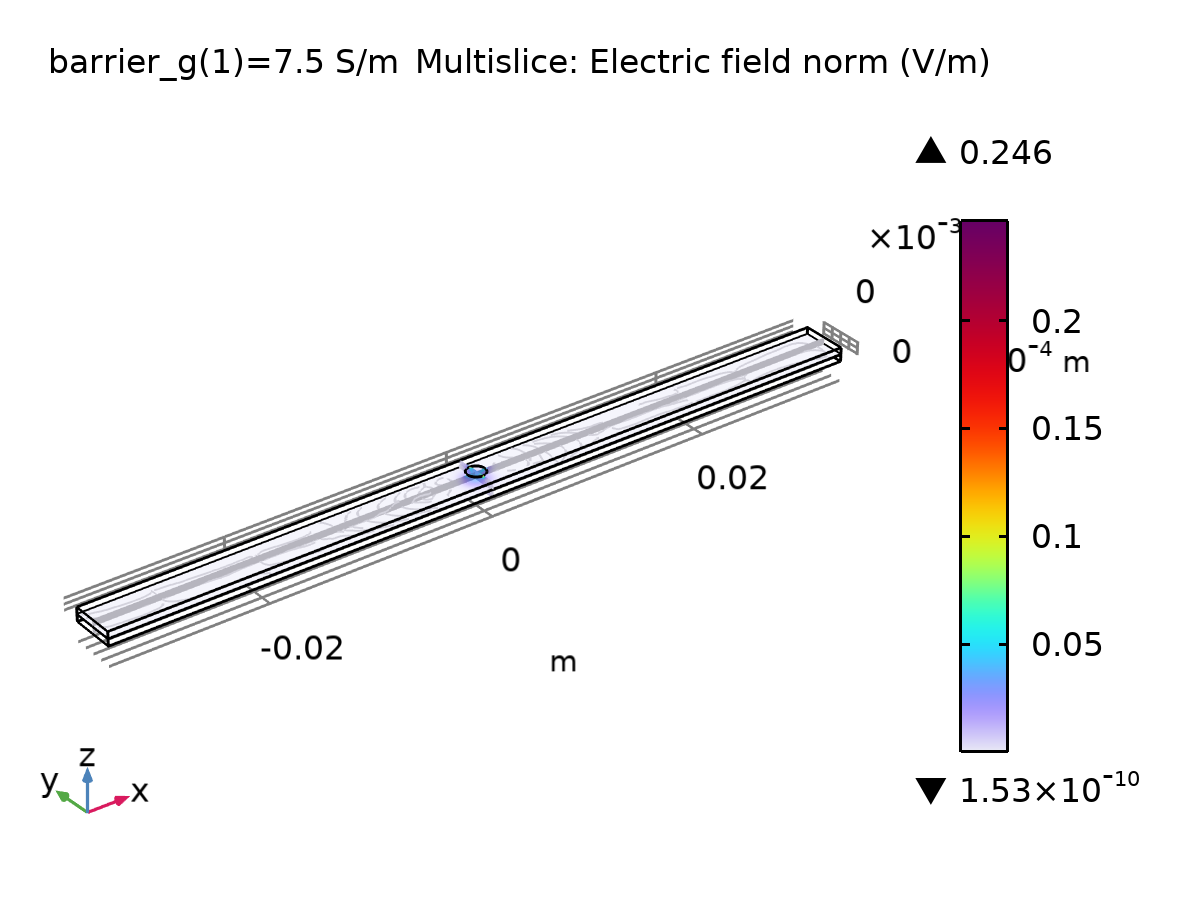


Multislice: Electric field norm (V/m)

- - 1. TEER


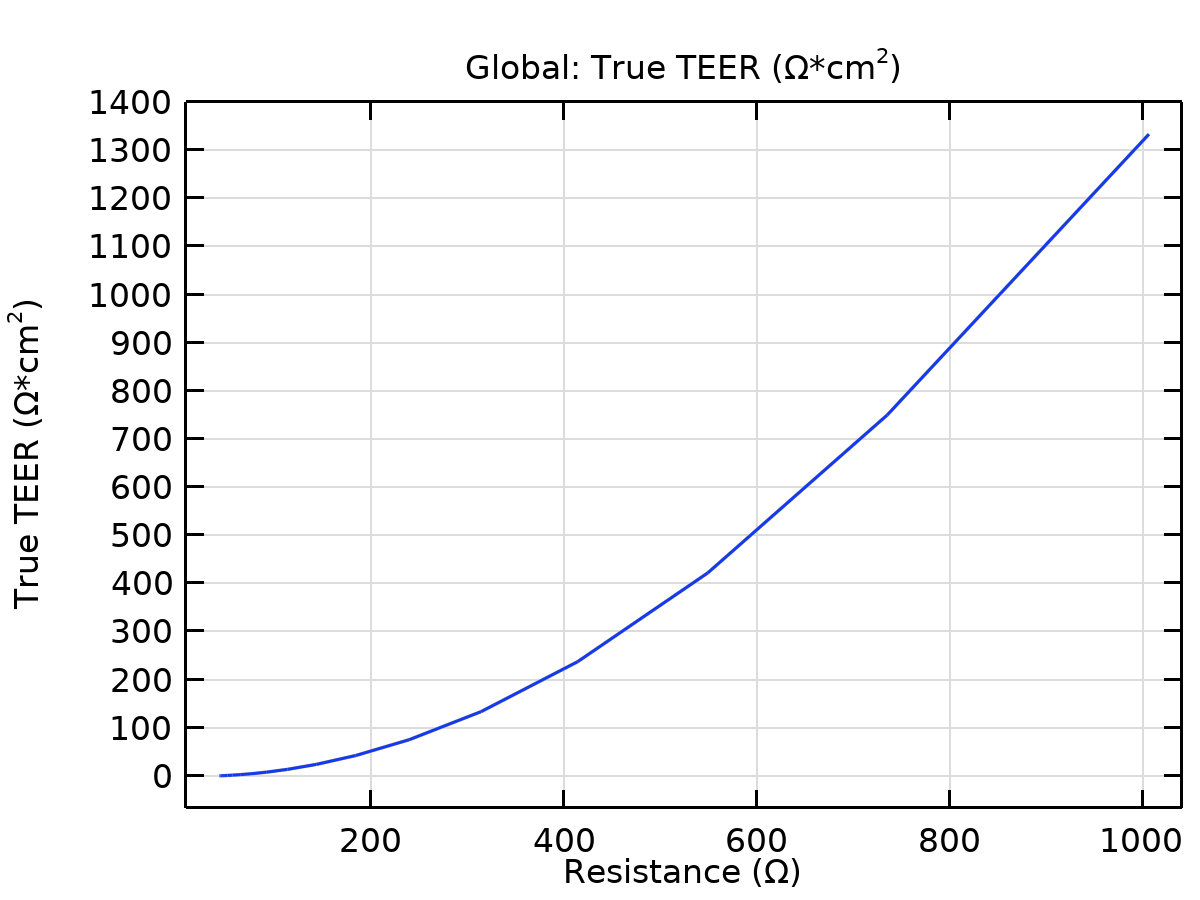


Global: True TEER (Ω*cm^2^)
