# Supplementary material for: Integration of multiple flexible electrodes for real-time detection of barrier formation with spatial resolution in a gut-on-chip system
Source: Microsyst Nanoeng. 2024 Jan 24;10:18. doi: 10.1038/s41378-023-00640-x (PMC10805851; doi:10.1038/s41378-023-00640-x)
Supplement: Supplementary file 2 — Supplementary Information [file 41378_2023_640_MOESM2_ESM.pdf]

Supplementary Information

Integration of multiple flexible electrodes for real-time detection of barrier formation with spatial resolution in a gut-on-chip system

Mara Lucchetti[1,†], Gabriel Werr[2,†], Sofia Johansson[2,*], Laurent Barbe[2], Léa Grandmougin[1], Paul Wilmes[1,3] and Maria Tenje[2]

[1] Luxembourg Centre for Systems Biomedicine (LCSB), University of Luxembourg, Esch-sur-Alzette, Luxembourg, L-4362

[2] Division of Biomedical Engineering, Department of Materials Science and Engineering, Science for Life Laboratory, Uppsala University, Uppsala, Sweden, 751 21

[3] Department of Life Sciences and Medicine, Faculty of Science, Technology and Medicine, University of Luxembourg, Esch-sur-Alzette, Luxembourg, L-4362

† These authors contributed equally

* Corresponding authors: Sofia Johansson (sofia.m.johansson@angstrom.uu.se)

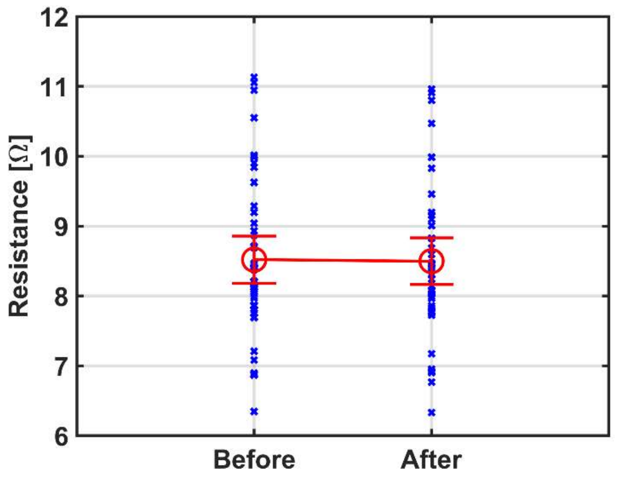

Figure S1: *Sheet resistance measurements on a 20x20 mm² electrode area prepared to evaluate the effect of transferring the tape between the carrier foil and the target device. A total of five samples were analysed with 9 4-point probe measurements collected from each, distributed over the sample area. The errorbars represent the standard error over all 45 measurement sites (n =45). In a paired-sample t-test no significant change in sheet resistance was observed in a confidence interval of p=0.1.*

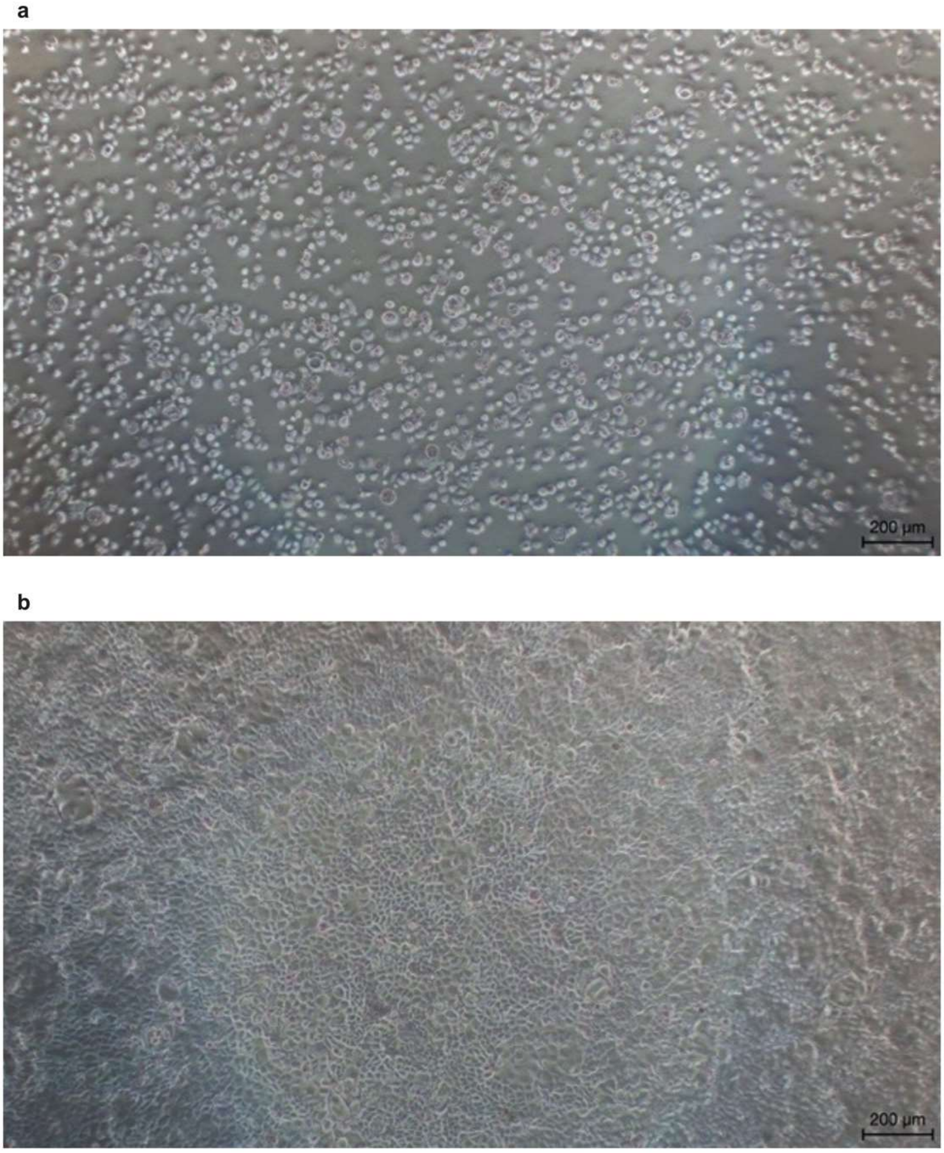

Figure S2: *Caco-2 cell shape. a) Cell shape two hours after seeding. b) Cell shape one week after seeding. Cells were imaged using a ZEISS Axiovert 40C inverted phase contrast microscope.*

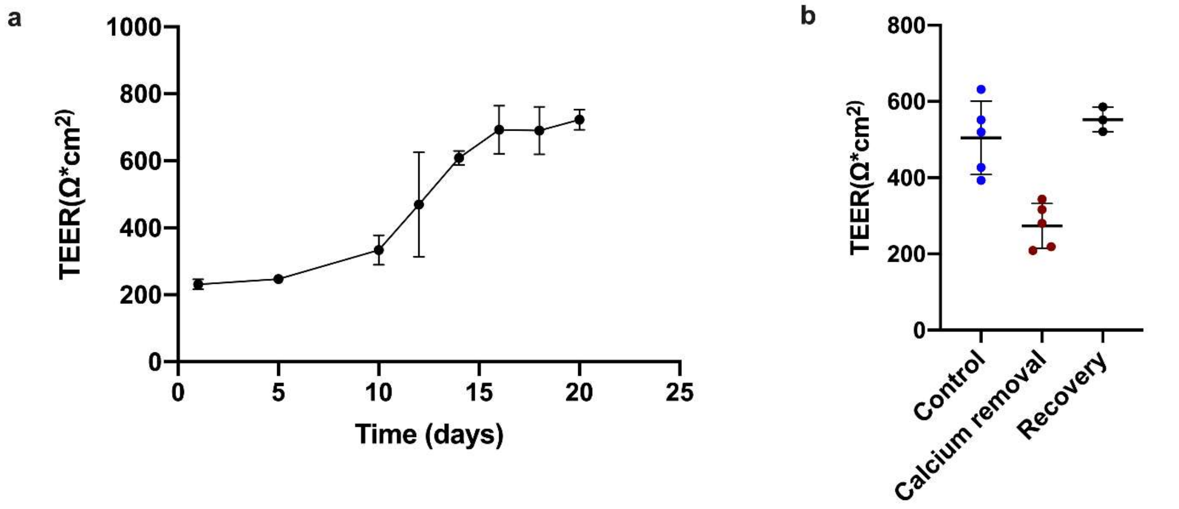

Figure S3: *TEER measured in a Transwell with chop-stick electrodes. a) The Caco-2 cells form a tight barrier in the 12-well Transwells after approx. two weeks. b) By removing calcium from the media, the TEER drops significantly (measured 1 hr after calcium removal). By exchanging the calcium-free media to standard cell culture media (RPMI), the TEER values recovered after 24 hr. Mean +/- SD of three to five independent replicates is shown.*

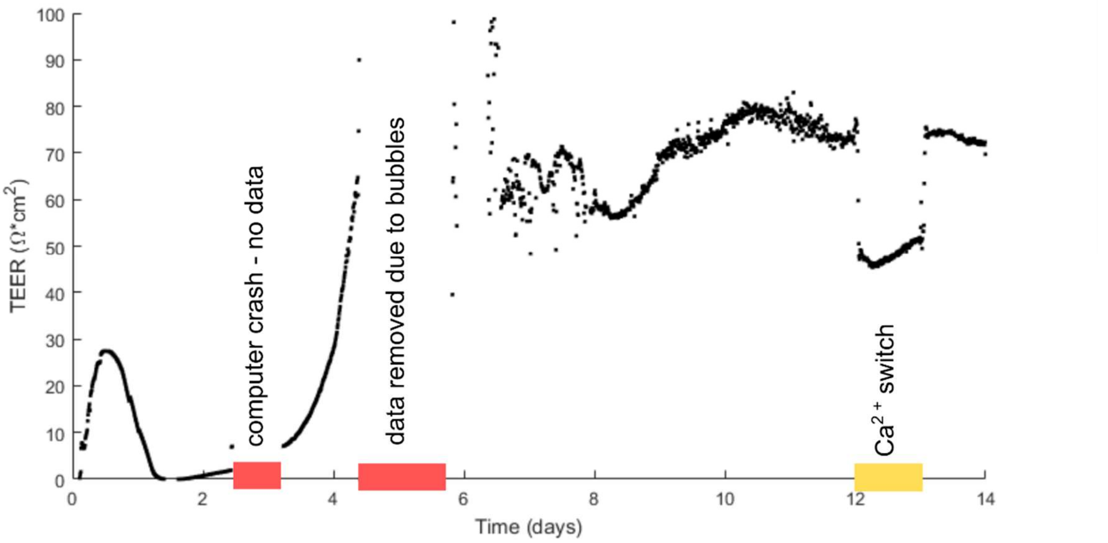

Figure S4: *One example of TEER values as a function of time in the HuMiX for all measured time points at which no bubbles were identified as a change of the high frequency impedance magnitude of more than 200 Ω compared to initial cell media only measurements. The measurements were taken at electrode position 2 (~29 mm from the inlet of the device) and normalised using the geometrical correction factor. D0 indicates the time at which cells were seeded in the chip. The red boxes indicate missing data. At the first occasion, there was an issue with the computer, leading to a stop in the automstic data collection. At the second occasion, bubbles were identified in the post-processing of the data. The yellow box indicates the time of the* Ca^{2+} *switch experiment.*

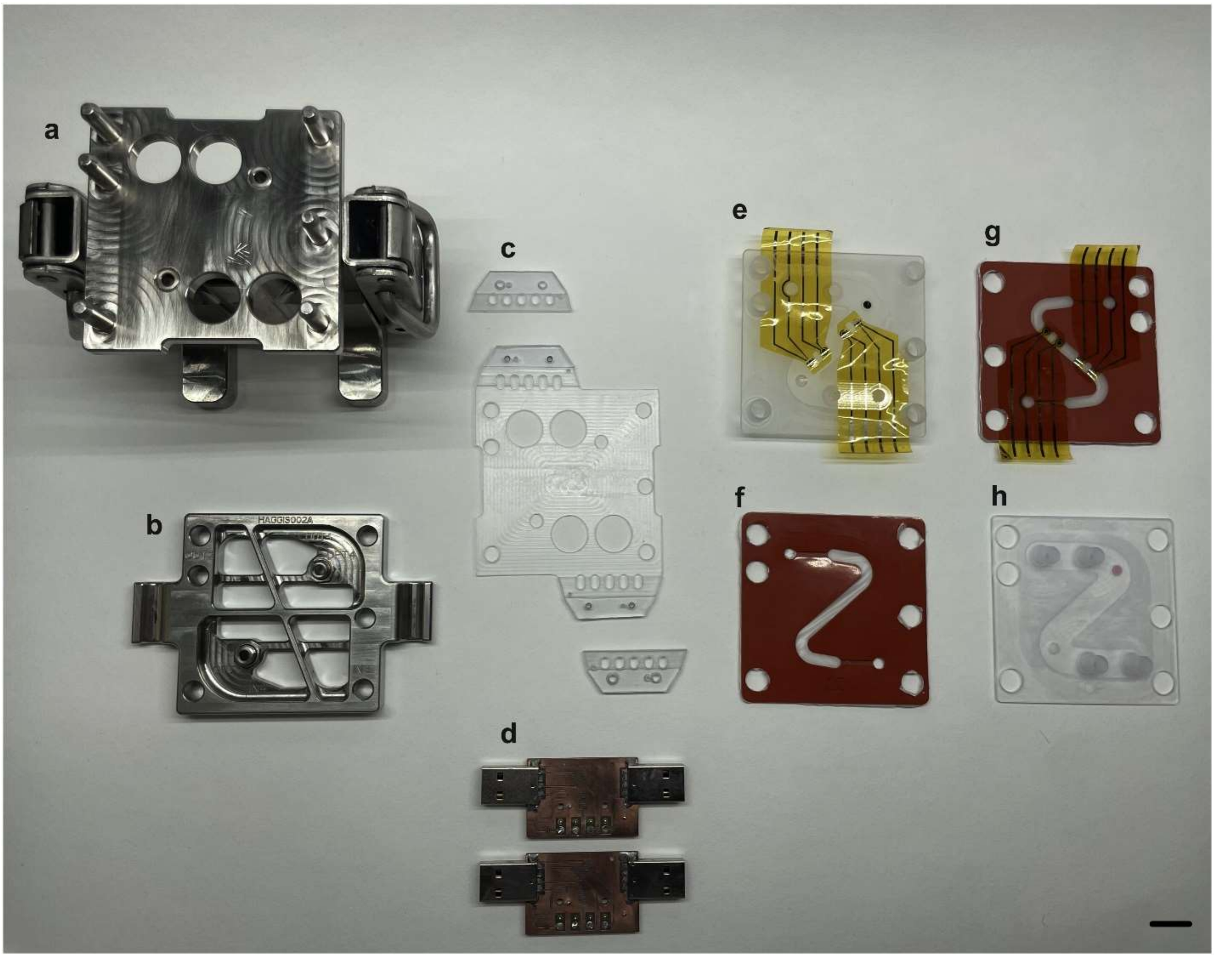

Figure S5: *Parts assembled to form the HuMiX platform. a) Metal base, b) Metal top part, c) PCB support and alignment pins, d) PCBs with pogo-pins, e) Bottom PC lid with electrodes, f) Gasket separating perfusion and epithelial chamber, g) Gasket with electrodes harbouring the bacterial chamber and separating it from the epithelial chamber on one side and the* N_2 *chamber on the other side, h) Top PC lid (scale bar = 1 cm).*

Table S1 *Parameters and equations used for the COMSOL simulation and post-processing to give the relationship between the measured resistance and true TEER and calculation of the geometrical correction factor.*

**Parameter**	**Value/equation**	**Comment**
Channel height, h	0.611 mm	Assuming 30% compression
Channel length, l	67.5 mm	
Channel width, w	4 mm	
Centre electrode width, w_{el}	1.6 mm	
Cell layer thickness, t_{cell}	0.010 mm	
Cell media conductivity, g_{media}	7.5 S/m	Giving reasonable background resistance
Cell barrier conductivity, g_{cell}	$7.5x10^{-5}$ - 7.5 S/m	Swept parameter
Cell culture area, A	2.7 cm^2	Used for calculations of apparent TEER
True TEER, *tTEER*	$tTEER = t_{cell}/g_{cell}$	
Apparent TEER, *aTEER*	$aTEER = (R-R_{bg})\cdot A$	R is obtained from the simulation, R_{bg} = 42.2 Ω is estimated as R at *tTEER* = 0.013 Ω·cm^2
Geometrical correction factor, *GCF*	$GCF = tTEER/aTEER$	

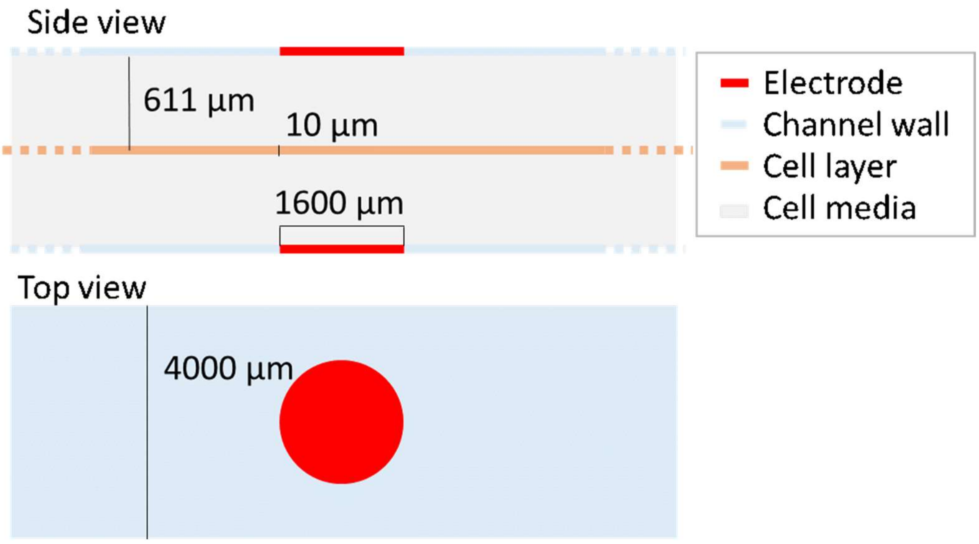

Figure S6: *Schematic of the geometry used for the COMSOL simulations for the relationship between the measured resistance and true TEER and calculations of the geometrical correction factor*

a

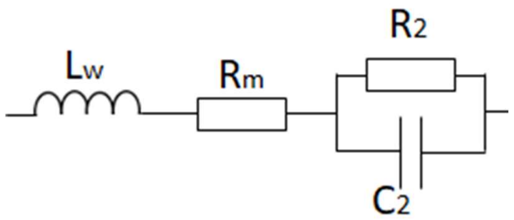

b

$$error = \frac{1}{n_f} \Sigma_f \frac{\left|1/Z_{model(f)} - 1/Z_{measured(f)}\right|^2}{\left(1/\left|Z_{measured(f)}\right|\right)^2}$$

c

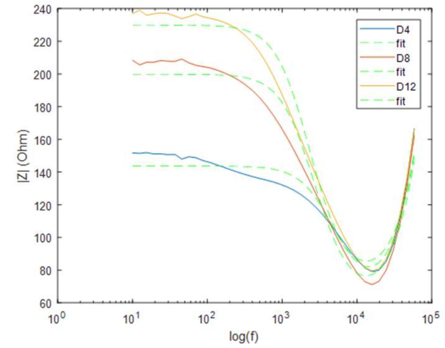

d

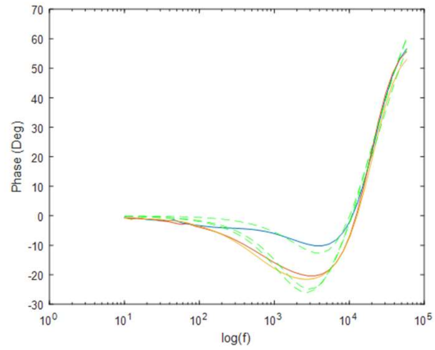

Figure S7: *a) Circuit model used to deduce TEER from the impedance spectra: Lw – inductance of wires, Rm –resistance of cell media, R2 – resistance of tight junction (TEER), and C2 – capacitance of cell membrane. b) The model was optimized by numerically minimizing the error of the admittance between the measured and modelled data using Matlab, where nf is the number of frequencies, f is the frequency,* Z_{model} *is the modelled impedance at f and* $Z_{measured}$ *is the measured impedance at f. c)-d) Measured impedance data from the first experiment (second position from the inlet) is compared with the optimized modelled data at D4, D8 and D12 after seeding the impedance magnitude in c) and the phase in d).*
